# Long-term performance evaluation of a 1.5T MR-Linac using statistical process control techniques

**DOI:** 10.1186/s13014-025-02670-3

**Published:** 2025-06-07

**Authors:** Qing Xiao, Mengdie Shen, Guangjun Li, Shipai Zhu, Jinrong He, Qiang Wang, Guyu Dai, Hang Yu, Jialu Lai, Renming Zhong, Sen Bai

**Affiliations:** 1 Department of Radiation Oncology, Cancer Center, West China Hospital, Sichuan University, No.37 Guoxue Alley, Chengdu, 610041 Sichuan China; 2 Department of Radiotherapy Physics & Technology, West China Hospital, Sichuan University, No.37 Guoxue Alley, Chengdu, 610041 Sichuan China; 3 Division of Abdominal Tumor Multimodality Treatment, Cancer Center, West China Hospital, Sichuan University, No.37 Guoxue Alley, Chengdu, 610041 Sichuan China

**Keywords:** MR-Linac, Statistical process control, Quality assurance, Adaptive radiotherapy, Geometric distortion

## Abstract

**Background:**

The integration of magnetic resonance imaging with linear accelerators (Linacs) enhances adaptive radiotherapy by providing real-time imaging for improved treatment precision. However, the long-term performance of MR-Linac systems, particularly in clinical settings, remains insufficiently studied. Traditional quality assurance (QA) methods, relying on binary pass/fail criteria, may overlook critical system variations. This study applies statistical process control (SPC) techniques to evaluate the long-term performance of a 1.5T MR-Linac, focusing on optimization in beam quality, MR-to-MV alignment, MR imaging, and geometric distortion.

**Methods:**

A dual-phase SPC framework was applied to 1 year of daily and weekly QA data from an Elekta Unity MR-Linac. Phase I established performance benchmarks, while Phase II monitored deviations online. Evaluated parameters included beam output, symmetry, MR-to-MV alignment, signal-to-noise ratio (SNR), spatial linearity, slice profile, and geometric distortion across spherical volumes (DSVs). Stability and variability were quantified using control charts and process performance indices (Ppk).

**Results:**

Beam quality was stable overall (Ppk ≥ 1.33), though output dose and transverse symmetry showed increased variability in Phase II, with dose Ppk declining from 3.13 to 1.33. MR-to-MV alignment was consistent, but Phi rotational and Z translational offsets showed variability after system upgrades. Imaging metrics, including SNR and spatial linearity, achieved A + performance (Ppk ≥ 1.67) in Phase II, while vertical spatial resolution was lower (Ppk 1.04–1.10). Geometric distortion was well-controlled, though larger DSVs (≥ 500 mm) showed increased AP-axis distortion (2.44 mm) compared to RL (1.37 mm) and FH (0.93 mm).

**Conclusions:**

SPC techniques dynamically identified stable parameters and areas for improvement. Key recommendations include enhanced alignment protocols for beam quality and MR-to-MV offsets, as well as targeted strategies to address geometric distortion in larger volumes and along the AP axis.

**Supplementary Information:**

The online version contains supplementary material available at 10.1186/s13014-025-02670-3.

## Introduction

The magnetic resonance linear accelerator (MR-Linac) integrates high-field (1.5T) magnetic resonance imaging (MRI) with a precision radiotherapy system, enabling real-time imaging and adaptive treatment planning [1]. This combination offers unparalleled advantages in soft-tissue visualization and treatment accuracy, making it a cornerstone of modern adaptive radiotherapy (ART). However, ensuring the system’s reliability and stability in clinical practice requires robust quality assurance (QA) protocols [2, 3].

The integration of MR with a Linac introduces unique QA challenges [4]. The high-field magnetic environment can affect beam characteristics, compromising dose accuracy and consistency [5, 6], while Linacs may further degrade magnetic field uniformity, causing image artifacts [7]. Achieving high-quality MR images, with stable signal-to-noise ratio (SNR), accurate scaling, and minimal geometric distortion, is essential for adaptive treatment planning [8, 9]. Routine QA must evaluate both imaging quality and geometric accuracy to ensure clinical efficacy [4, 8].

Despite the critical importance of QA for MR-Linac systems, existing protocols remain insufficiently established, creating challenges in ensuring consistent performance across clinical implementations [2]. Traditional QA approaches, predominantly based on binary pass/fail criteria, offer simplicity but lack the sensitivity to detect subtle performance trends or gradual deviations in key metrics, such as beam characteristics or imaging quality, especially over extended periods [10, 11]. This limitation not only restricts their effectiveness in long-term performance monitoring but also highlights the need for advanced and dynamic QA methodologies tailored to the unique complexities of MR-Linac systems [11, 12].

Statistical process control (SPC) is a well-established tool in quality management and has recently gained attention in radiotherapy QA [10, 13–15]. Unlike conventional methods, SPC enables dynamic monitoring of process stability and provides deeper insights into system performance [10, 16]. Specifically, SPC offers three key advantages: (1) it detects minor process variations that binary methods may overlook; (2) it evaluates the current state of the process through control charts, allowing for proactive intervention; and (3) it supports continuous improvement using performance metrics such as process capability indices or process performance indices. The application of SPC to MR-Linac QA offers a promising approach to overcome limitations of traditional QA methods and ensure consistent performance over time [12].

This study applies a comprehensive SPC framework to assess the long-term performance stability of an Elekta Unity MR-Linac over a 1-year clinical operation period. Daily and weekly QA data, including key metrics such as beam consistency, MR-to-MV isocenter alignment, MR image quality parameters, and geometric distortion, were systematically analyzed. A dual-phase SPC framework was adopted: Phase I involved retrospective baseline analysis to establish performance benchmarks, while Phase II focused on real-time monitoring to detect deviations and maintain stability. The study aims to (1) ensure the MR-Linac achieves the high precision required for adaptive radiotherapy and (2) identify critical QA parameters for process optimization. These efforts contribute to the development of standardized QA protocols and support the integration of enhanced QA practices into routine clinical workflows.

## Materials and methods

This study investigates the long-term stability of an Elekta Unity MR-Linac (see Fig. A1a in Additional file 1 for details) through daily and weekly QA data analysis using SPC techniques. Monthly and annual QA tests, being less frequent, were excluded from the scope of this study. The QA program comprises four core components: (1) beam quality checks, (2) MR-to-MV isocenter alignment, (3) MR image quality evaluations, and (4) MR geometric distortion analysis. A detailed summary of the QA tests, including their associated phantoms, properties tested, and measurement frequency, is presented in Table 1. The coordinate system follows the IEC 61217 standard for radiation therapy equipment positioning [17].

**Table 1:** Overview of QA tests and measurement parameters for MR-Linac systems

Test	Device	Subsystem	Tested Properties	Frequency
Beam quality checks	Sun Nuclear Daily QA-MR	Linac (Gantry 0° and 180°)	Energy, output, transverse and axial symmetry, shape constancy, and field size shift	Daily
MR-to-MV concordance	Elekta MR-to-MV phantom	MR and MV radiation isocenters	Translation offset (X、Y、Z); Rotation offset (Psi、Phi、Theta)	Daily
MRL_QA_SNR	Philips 200 mm head Phantom	Marlin anterior and posterior coils	SNR, uniformity	Daily
MRL_QA_SCALING	Philips 200 mm head Phantom	Marlin anterior and posterior coils	Small FOV spatial scaling	Daily
Periodic image quality test (PIQT)	Philips 200 mm head Phantom	All Marlin coils	SNR, uniformity, spatial linearity, slice profile, spatial resolution	Daily
3D geometric QA	3D geometric QA Phantom	Marlin system body coil	Large FOV geometric distortion	Weekly

MR = magnetic resonance; MV = megavoltage; SNR = signal-to-noise ratio; FOV = field of view; QA = quality assurance; Linac = linear accelerator

### Beam quality checks

Beam quality checks were performed to assess the stability of the Linac output using the MR-compatible Daily QA-MR device (DQA, Sun Nuclear Corporation, Melbourne, FL), a system widely recognized for its application in MR-Linac QA [2, 3, 18], as illustrated in Fig. A1-b of Additional File 1. To account for the unique characteristics of the MR-Linac’s ring-shaped structure, measurements were conducted at both gantry angles of 0° and 180°, unlike conventional DQA protocols that typically assess only at 0°. Daily QA data were systematically analyzed to evaluate short-term variations and long-term trends, focusing on key metrics such as output consistency and symmetry (summarized in Table 1).

### MR-to-MV concordance

The MR-to-MV alignment tests assessed the spatial congruence between MR and MV isocenters using the Elekta MR-to-MV QA phantom (Fig. A1-e in Additional File 1), containing seven ceramic spheres immersed in copper sulfate [3, 18, 19]. Simultaneous acquisition of MV images at 10 predefined gantry angles (e.g., 0°, 60°, 78°) and a T1-weighted MR image was performed [18]. Translational (X, Y, Z) and rotational (Psi (ψ), Phi (φ), Theta (θ)) offsets—defined according to the IEC 61217 coordinate system—were calculated using vendor-provided QA alignment software to evaluate deviations relative to the baseline established during commissioning. This test ensured that alignment deviations remained within the clinical tolerance of 1.0 mm [18], ensuring precise imaging and treatment delivery.

### MR image quality tests

Daily MR image quality was assessed using the Philips 200 mm head phantom (Fig. A1-c and d in Additional File 1) with the vendor-provided system performance tool (SPT) for automated image acquisition and analysis [3, 20]. Three key tests performed were: (1) periodic image quality test (PIQT): Evaluates parameters such as signal-to-noise ratio (SNR), uniformity, spatial linearity (SPL), spatial resolution (SPR), and slice profile (SLP); (2) SNR Test: Measures element-wise SNR for the Marlin anterior and posterior coils; (3) Scaling Test: Verifies geometric accuracy in transverse and coronal planes within a small FOV. The phantom was positioned horizontally for coronal scaling and vertically for other tests. For details, see Subashi E et al. [20].

The PIQT test does not assess the element-wise SNR or coronal scaling. Although the SNR and scaling tests partially overlap with PIQT, all tests were conducted daily to ensure comprehensive monitoring of imaging system performance. A total of 70 imaging parameters were analyzed to ensure consistency and accuracy.

### MR geometric distortion tests

Geometric distortion tests were conducted weekly using a 3D geometric QA phantom with 1932 grid markers [8, 20]. The phantom consisted of fiducial markers arranged in a precisely machined grid (Fig. A1-f in Additional File 1). Geometric distortion was calculated as the deviation between the measured and known marker positions. Distortion values were monitored across spherical volumes of varying diameters (200, 300, 400, and 500 mm). These tests ensured geometric fidelity, crucial for accurate target delineation during treatment planning.

### Data processing and SPC analysis

QA data were collected from the Elekta Unity MR-Linac system at West China Hospital. A Python-based framework was developed to automate data extraction, processing, and analysis. SPC methods were implemented to monitor system performance and detect deviations over time. Correlation analysis was performed to assess the geometric accuracy between the PIQT and Scaling Test. Pearson correlation coefficients were calculated and visualized in heatmaps.

#### SPC framework

Individual (I) Charts and Individual-Moving Range (I-MR) Charts are among the most commonly used control charts for monitoring processes with single observations, as described in Montgomery’s textbook [21]. In this study, individual control charts were constructed using a weighted standard deviation (WSD)-based method, which is well-suited for both normal and non-normal distributions [22, 23]. Key equations for the analysis are as follows:

1 $${MR}_{i}=\left|{X}_{i}-{X}_{i-1}\right|$$

2 $$\overline{MR }=\frac{\sum_{i=2}^{N}{MR}_{i}}{N-1}$$

3 $${CL}_{X}=\overline{X }=\frac{\sum_{i=1}^{N}{X}_{i}}{N}$$

4 $$P_{X} = \frac{{\mathop \sum \nolimits_{i = 1}^{N} I\left( {\overline{X} - X_{i} } \right)}}{N},\;I\left( x \right) = \left\{ {\begin{array}{*{20}c} {1,x \ge 0} \\ {0,x < 0} \\ \end{array} } \right.$$

5 $${LCL}_{X}=\overline{X }-\frac{3\cdot \overline{MR}}{{d }_{2}^{WSD}}{\cdot 2\left(1-P\right.}_{X})$$

6 $${UCL}_{X}=\overline{X }+\frac{3\cdot \overline{MR}}{{d }_{2}^{WSD}}{\cdot 2P}_{X}$$

Here, ${MR}_{i}$ quantifies the variability between consecutive data points. The central line (${CL}_{X}$) corresponds to the process mean ($\overline{X }$). The proportion of positive deviations (${P}_{X}$) captures asymmetry in the data distribution, while the lower (${LCL}_{X}$) and upper (${UCL}_{X}$) control limits incorporate weighted variability to detect process trends and deviations. The control chart constant (${d}_{2}^{WSD}$) is used to reliably estimate process variability, ensuring accurate calculation of control limits [22, 23]. Considering the impact of sampling variability on the performance of I-charts, a median-based tolerance limit method previously reported in the literature [24] was adopted.

This WSD-based SPC framework was implemented in two phases. Phase I used historical QA data from the first 8 months to establish control limits and achieve statistical control through an iterative refinement process [23, 24]. For each evaluation metric, 1000 independent random samples were generated through random sampling of the Phase I data. Within each sampled dataset, out-of-control points were identified and removed, and the lower and upper control limits (LCL and UCL) were recalculated iteratively until statistical control was achieved. The final control limits for each metric were defined as the median values of the 1000 resulting LCLs and UCLs. A detailed example of the control limit calculation process is provided in Additional file 2.

All original Phase I data points, including initially identified outliers, were retained when constructing the control charts, although out-of-control points were excluded during control limit calculation. This approach preserves the original data structure and provides a complete representation of the process variability observed during Phase I. In Phase II, the control limits established during Phase I were applied to real-time QA data to monitor for potential process shifts and deviations over time. This two-phase framework enables systematic tracking of performance stability in clinical applications.

#### Process performance analysis

The process performance index (${P}_{pk}$) was calculated to evaluate the long-term performance of the MR-Linac system. For dual-sided specification limits, ${P}_{pk}$ was determined using the formula [25]:

7 $$P_{pk} = \min \left\{ {\frac{{\overline{X} - LSL}}{{6\left( {1 - P_{X} } \right)s}}\frac{{USL - \overline{X}}}{{6P_{X} s}}} \right\}$$

Here, $\overline{X }$ is the process mean, $s$ is the standard deviation, ${P}_{X}$ is the proportion of positive deviations, and $LSL$ and $USL$ are the lower and upper specification limits, respectively. For single-sided specifications, the corresponding calculation used either $\frac{{\overline{X} - LSL}}{{6\left( {1 - P_{X} } \right)s}}$ or $\frac{{USL - \overline{X}}}{{6P_{X} s}}$, depending on the specification orientation.

Based on ${P}_{pk}$, process performance was graded into four levels (Table 2), ranging from optimal (“A + ”) to poor (“C”). Grades “A + ” and “A” were deemed optimal or acceptable, while grades “B” and “C” indicated moderate risk or poor performance, requiring corrective action. The specification limits were primarily derived from the Elekta MR-Linac consortium report [2] and vendor-provided reference data. In addition, although most capability classifications reported in the literature refer to Cp or Cpk indices, ${P}_{pk}$ values follow similar interpretative principles for assessing long-term process performance. Therefore, the thresholds for ${P}_{pk}$-based classification (1.00, 1.33, and 1.67) in this study were determined by analogy to these established criteria [21, 26–29].

**Table 2:** Process capability grading based on Ppk

Grade	Ppk value	Process description
A +	Ppk ≥ 1.67	Optimal; exceeds specifications, high confidence
A	1.67 > Ppk ≥ 1.33	Good; meets specifications, maintain stability
B	1.33 > Ppk ≥ 1	Acceptable; moderate risk, improvement recommended
C	Ppk < 1	Poor; significant improvement required

Ppk = process performance index

## Results

A systematic evaluation of beam quality, MR-to-MV alignment, MR image quality, and 3D geometric distortion is presented below. To complement the main results and provide additional distributional insights, detailed statistical analyses—including skewness, kurtosis, and normality test results—are available in the supplementary material (Additional file 1, Tables A1–A4).

### Beam quality analysis

**Fig. 1:**
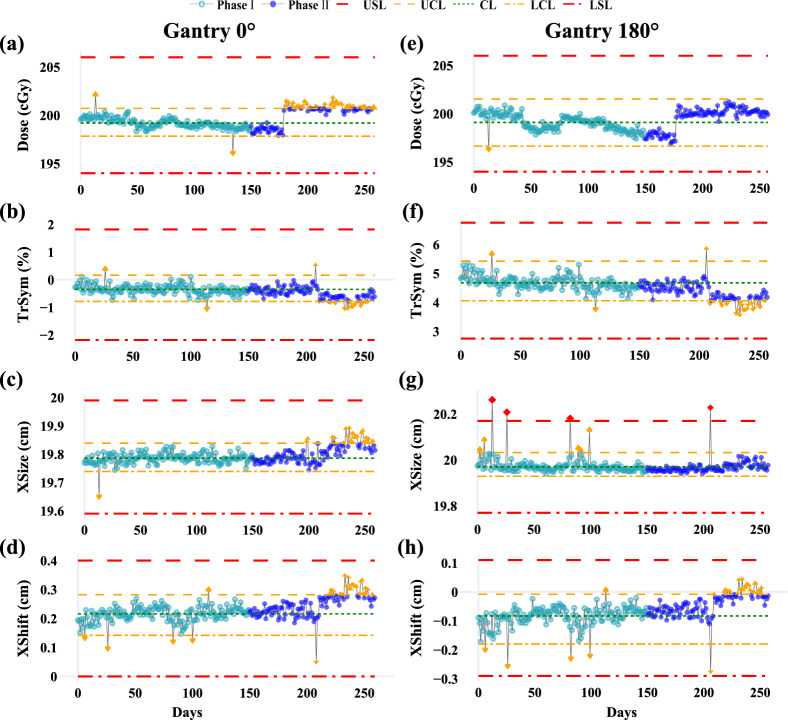
Control charts for beam quality parameters (**Dose**, **TrSym**, **XSize**, and **XShift**) at gantry 0° (**a**–**d**) and gantry 180° (**e**–**h**) during Phase I (light teal blue) and Phase II (blue). Red dashed lines indicate specification limits (LSL, USL), orange dashed and dash-dot lines represent control limits (UCL, LCL), and the green dotted line marks the central line (CL). OOC points are shown as orange triangles, and OOS points are red diamonds

This study assessed beam quality parameters at gantry angles 0° and 180°, including output dose (**Dose**), axial and transverse symmetry (**AxSym**, **TrSym**), energy stability (**Energy**), field size along the lateral (**XSize**) and longitudinal (**YSize**) axes, and beam positional shifts in the same directions (**XShift**, **YShift**). Key findings are presented in Table 3. **AxSym**, **Energy**, **YSize**, and **YShift** showed stable performance with Ppk > 1.33 and out-of-control (OOC) rates < 5.0%, indicating well-controlled processes. In contrast, **Dose**, **TrSym**, **XSize**, and **XShift** exhibited higher OOC rates (Fig. 1). A 1.5% underdosing detected during monthly QA necessitated recalibration, causing a shift in dose output at the 180th data point for both angles.

**Table 3:**
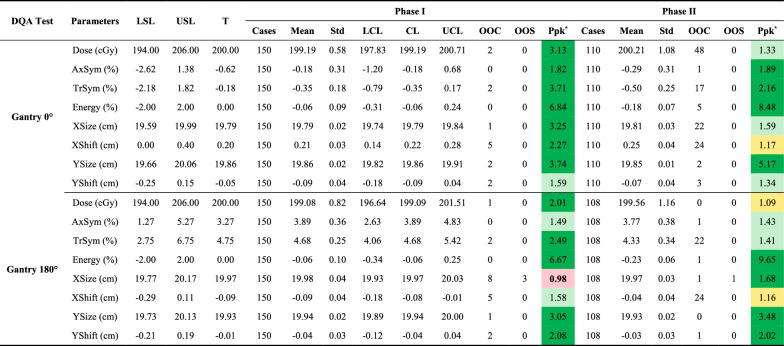
Summary of beam quality parameters from the DQA test at gantry 0° and 180°

DQA = daily QA; LSL = lower specification limit; USL = upper specification limit; T = baseline (target) value; Std = standard deviation; LCL = lower control limit; CL = central line; UCL = upper control limit; OOC = out-of-control cases; OOS = out-of-specification cases; Ppk = process performance index; AxSym = axial symmetry, TrSym = transverse symmetry; XSize = beam size along the X axis; XShift = beam shift along the X axis; YSize = beam size along the Y axis; YShift = beam shift along the Y axis

^*^: Color coding for Ppk: Green: A + (Ppk ≥ 1.67); Light green: A (1.67 > Ppk ≥ 1.33); Yellow: B (1.33 > Ppk ≥ 1.00); Light red: C (Ppk < 1.00)

Performance declined notably for **Dose** and **TrSym** in Phase II. At gantry 0°, **Dose** Ppk dropped from 3.13 to 1.33, with OOC rates rising to 43.6%. At gantry 180°, while no OOC points were observed, **Dose** Ppk fell from 2.01 to 1.09, reducing its rating from A + to B. **TrSym** variability increased at both angles, with OOC rates exceeding 15.4%. Positional parameters showed similar trends; for gantry 0°, **XShift** Ppk fell from 2.27 to 1.17, and OOC rates rose to 21.8%. **XSize** showed reduced stability at gantry 0° but improved at gantry 180°, with Ppk increasing from 0.98 to 1.68 and OOC rates declining to 0.9%. A single out-of-specification (OOS) event at gantry 180° was attributed to a setup error. The observed trends in **TrSym** and **XSize** fluctuations aligned with the increased variability in **XShift** during Phase II (Fig. 1).

### MR-to-MV concordance analysis

**Fig. 2:**
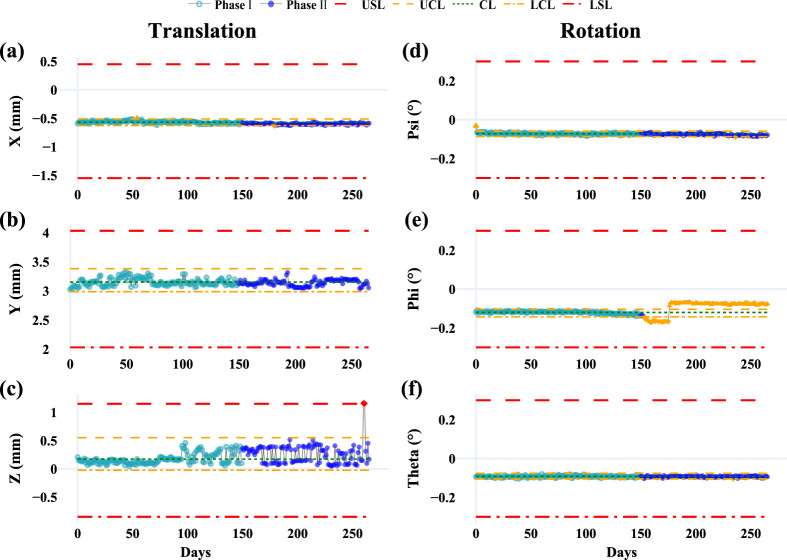
Control charts for MR-to-MV alignment parameters during Phase I (light teal blue) and Phase II (blue). Translation parameters are shown in **a** X, **b** Y, and **c** Z, while rotation parameters are shown in **d** Psi, **e** Phi, and **f** Theta. Red dashed lines indicate specification limits (LSL, USL), orange lines (dashed and dash-dot) represent control limits (UCL, LCL), and the green dotted line marks the central line (CL). OOC points are shown as orange triangles, and OOS points are red diamonds

Table 4 summarizes the MR-to-MV alignment performance, evaluating rotational (Psi, Phi, Theta) and translational (X, Y, Z) offsets. All parameters maintained A + performance (Ppk ≥ 1.80) except for the Phi rotational offset in Phase II, where performance declined to B (Ppk = 1.21). Control charts are shown in Fig. 2.

**Table 4:**
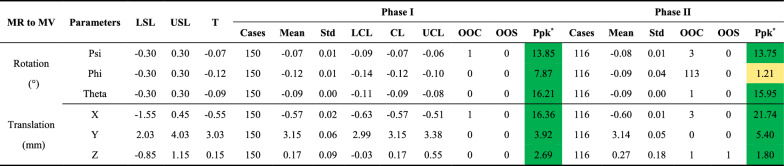
Summary of MR-to-MV alignment performance for rotational and translational offsets during Phase I and Phase II

MR to MV: Magnetic resonance to megavoltage alignment; LSL = lower specification limit; USL = upper specification limit; T = baseline (target) value; Std = standard deviation; LCL = lower control limit; CL = central line; UCL = upper control limit; OOC = out-of-control cases; OOS = out-of-specification cases; Ppk = process performance index

^*^: Color coding for Ppk: Green: A + (Ppk ≥ 1.67); Light green: A (1.67 > Ppk ≥ 1.33); Yellow: B (1.33 > Ppk ≥ 1.00); Light red: C (Ppk < 1.00)

In Phase I, translational offsets along X, Y, and Z were well-controlled, with only one OOC point in the X direction. Rotational offsets also remained stable, except for one OOC point in Psi. These results indicate consistent MR-to-MV spatial alignment during Phase I.

Phase II, however, showed reduced performance. While the Y translational offset remained stable, other parameters exhibited increased variability. The Phi rotational offset notably deteriorated, with 86% of data points out-of-control, lowering its Ppk to B. Variability also reduced the Z rotational offset’s Ppk from 2.69 to 1.80, aligning with the fluctuations in Fig. 2. On the same day as the observed OOS event, restarting the Linac and Marlin MR system restored all parameters to normal. Additionally, increased variability in the Z translational offset was noted from data point 95 (Fig. 2c), coinciding with the Marlin MR system upgrade on March 9, 2024.

### MR image quality analysis

**Fig. 3:**
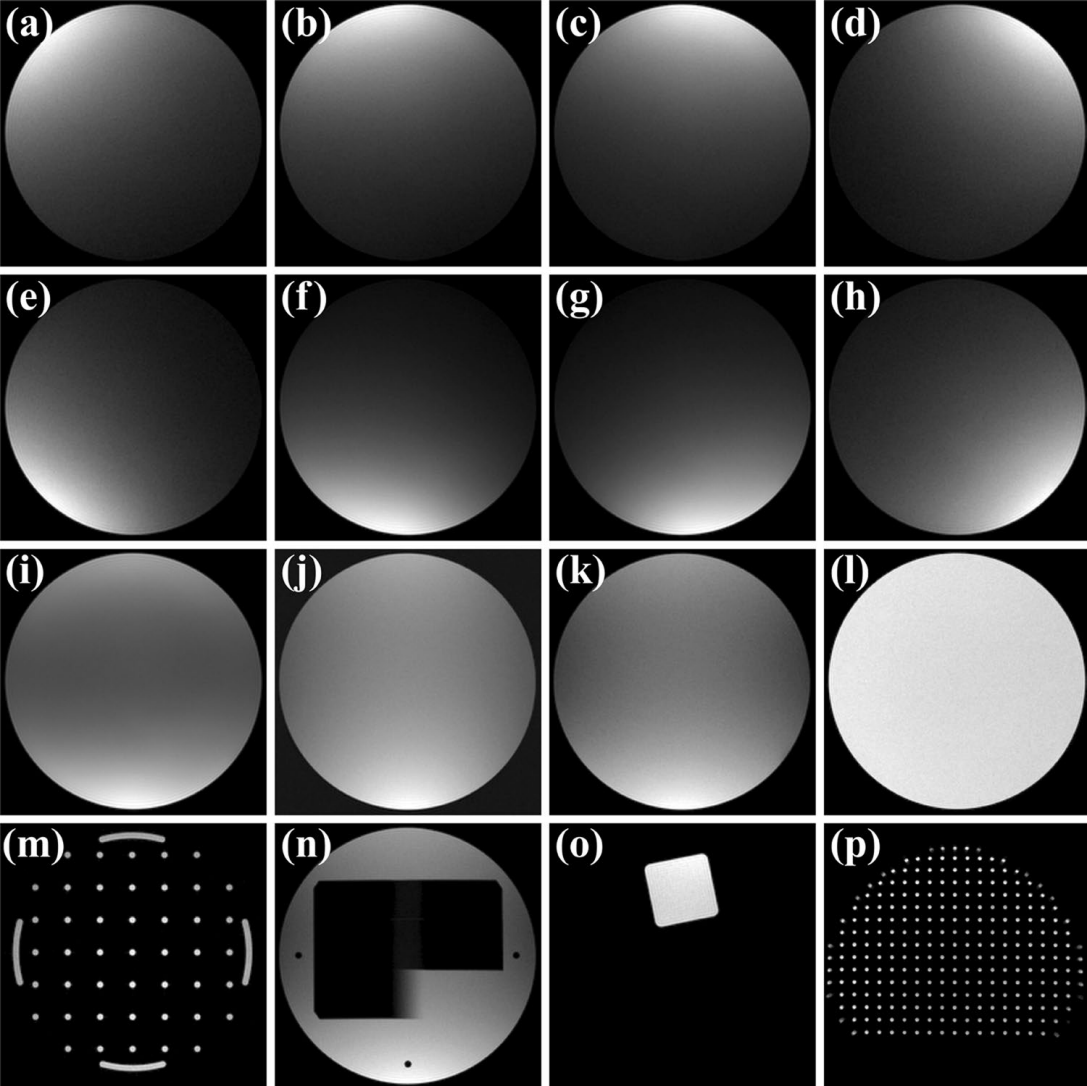
Representative MR images from quality tests. **a**–**i** Flood field uniformity (FFU) test images from the MRL_QA_SNR scans: **a**–**d** anterior coil elements (T_1–T_4), **e**–**h** posterior coil elements (T_5–T_8), and **i** combined result across all elements. **j**–**l** Uniformity test images from the PIQT scans: **j** QA1, Spin-echo (TE = 100 ms) using anterior and posterior coils; **k** QA2, Gradient-echo (TE = 15 ms) using anterior and posterior coils; **l** QA3, Spin-echo (TE = 100 ms) using the system body coil. **m** Spatial linearity test image from the MRL_QA_SCALING scan. **n** Slice profile test image from the PIQT scan. **o** Spatial resolution test image from the PIQT scan. **p** 3D geometric distortion test image

**Fig. 4:**
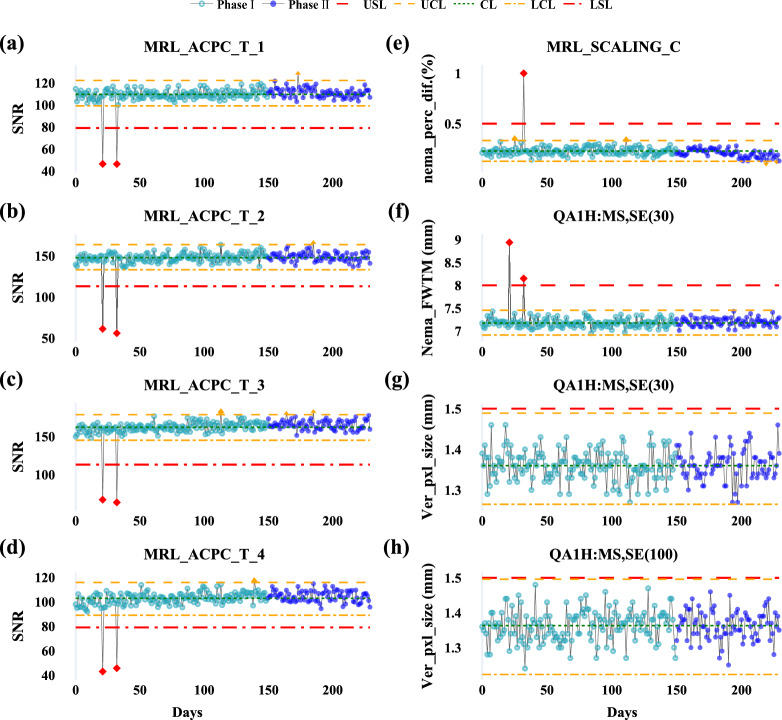
Control charts of MR image quality metrics with Ppk values at or below B level in Phase I (light teal blue) and Phase II (blue). Subfigures show: **a**–**d** SNR for four elements of the Marlin anterior coil, **e** spatial linearity (**nema_perc_dif.**) for SCALING_C scans, **f** FWHM for QA1H:MS scans, and **g**–**h** vertical pixel size for QA1H:MS scans at echo times of 30 ms and 100 ms. Red dashed lines indicate specification limits (LSL, USL), orange lines (dashed and dash-dot) represent control limits (UCL, LCL), and the green dotted line marks the central line (CL). OOC points are orange triangles, and OOS points are red diamonds

Representative MR images from quality tests are shown in Fig. 3. MR image quality metrics, including MRL_QA_SNR (Table 5), MRL_QA_SCALING (Table 6), and PIQT (Table 7), showed improved performance in Phase II compared to Phase I, with nearly all parameters remaining within specification limits and no OOS events observed. For the eight Phase I parameters with Ppk values at or below B level, control charts are presented in Fig. 4. In Phase I, two major OOS events were identified: one due to failure to lower the anterior coil to the phantom’s top and the other from leaving the ArcCHECK-MR phantom on the treatment couch after plan verification. Correcting these issues aligned Phase I variability with Phase II, as shown in Fig. 4a–f.

**Table 5:**
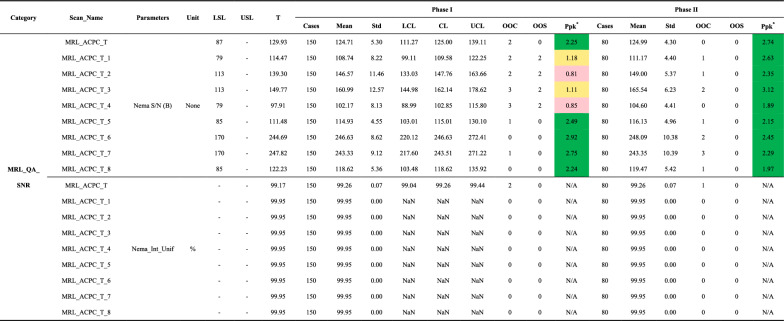
Summary of MRL_QA_SNR metrics for flood field uniformity tests, including signal-to-noise ratio (SNR) and image uniformity across Phase I and Phase II

SNR = signal-to-noise ratio; LSL = lower specification limit; USL = upper specification limit; T = baseline (target) value; Std = standard deviation; LCL = lower control limit; CL = central line; UCL = upper control limit; OOC = out-of-control cases; OOS = out-of-specification cases; Ppk = process performance index; MRL_ACPC_T (and subelements) = scans using all or specific elements of the Marlin Anterior/Posterior coil (e.g., T_1 = Anterior coil element 1; T_5 = Posterior coil element 1); NEMA_Int_Unif = integral uniformity; N/A = not applicable; NaN = not available; -: not provided

^*^: Color coding for Ppk: Green: A + (Ppk ≥ 1.67); Light green: A (1.67 > Ppk ≥ 1.33); Yellow: B (1.33 > Ppk ≥ 1.00); Light red: C (Ppk < 1.00)

**Table 6:**
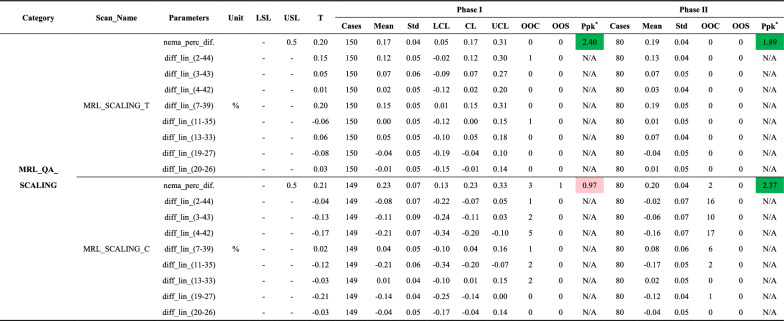
Summary of MRL_QA_SCALING metrics for spatial linearity tests, including the maximum absolute percentage deviations (**nema_perc_dif.**) and pairwise distance differences (**diff_lin_(x–y)**) across Phase I and Phase II

nema_perc_dif = NEMA-defined maximum absolute percentage deviation; diff_lin = pairwise distance difference; LSL = lower specification limit; USL = upper specification limit; T = baseline (target) value; Std = standard deviation; LCL = lower control limit; CL = central line; UCL = upper control limit; OOC = out-of-control cases; OOS = out-of-specification cases; Ppk = process performance index; N/A = not applicable; NaN = not available; -: not provided

^*^: Color coding for Ppk: Green: A + (Ppk ≥ 1.67); Light green: A (1.67 > Ppk ≥ 1.33); Yellow: B (1.33 > Ppk ≥ 1.00); Light red: C (Ppk < 1.00)

**Table 7:**
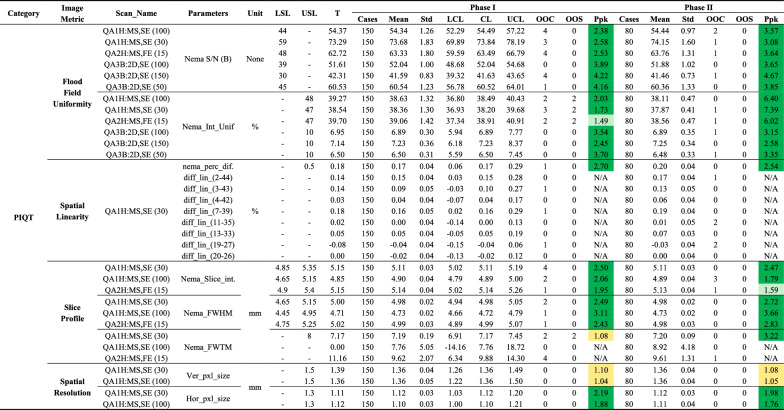
Summary of PIQT metrics for flood field uniformity, spatial linearity, slice profile, and spatial resolution tests across Phase I and Phase II

LSL = lower specification limit; USL = upper specification limit; T = baseline (target) value; Std = standard deviation; LCL = lower control limit; CL = central line; UCL = upper control limit; OOC = out-of-control cases; OOS = out-of-specification cases; Ppk = process performance index; SNR = signal-to-noise ratio; Nema_Int_Unif = integral uniformity; Nema_Slice_int = integral of slice profile; FWHM = full-width at half maximum; FWTM = full-width at tenth maximum; nema_perc_dif. = NEMA-defined maximum absolute percentage deviation; diff_lin_(x–y) = pairwise distance deviation; Ver_pxl_size = vertical pixel size; Hor_pxl_size = horizontal pixel size; QA1H:MS, SE = Marlin Anterior and Posterior coils with spin-echo sequences; QA2H:MS, FE = Marlin Anterior and Posterior coils with gradient-echo sequences; QA3B:2D, SE = System Body Coil (QBC) with spin-echo sequences; N/A = not applicable; NaN = not available; -: not provided

^*^: Color coding for Ppk: Green: A + (Ppk ≥ 1.67); Light green: A (1.67 > Ppk ≥ 1.33); Yellow: B (1.33 > Ppk ≥ 1.00); Light red: C (Ppk < 1.00)

#### Flood field uniformity

The flood field uniformity (FFU) test assessed SNR and uniformity using the phantom’s FFU section. In PIQT tests, SNR measurements from the MRL anterior/posterior coils (QA1, QA2) and the system body coil (QA3) consistently performed at an A + level across both phases, with no OOS events and a maximum OOC rate of 2.7% (Table 7).

In the MRL_QA_SNR test, Phase I revealed challenges with Ppk for four elements of the Marlin anterior coil falling to B level or below due to two OOS events (Table 5). Following corrective actions, Phase II showed full recovery, with all components achieving A + Ppk and a maximum OOC rate of 3.8%.

Uniformity measurements from the MRL_QA_SNR tests remained stable across both phases, closely aligning with baseline values and showing no significant deviations (Table 5). PIQT-based uniformity measurements with the system body coil consistently maintained A + Ppk throughout both phases (Table 7). Uniformity from the anterior/posterior coils, initially affected in Phase I, improved significantly in Phase II, highlighting the system’s ability to restore and sustain high-level performance following corrective actions.

#### Spatial linearity

Spatial linearity, which evaluates in-plane geometric distortion across eight radial directions (Fig. A2 in Additional File 1), was assessed using MRL_QA_SCALING and PIQT tests. Key metrics included **nema_perc_dif.**, representing maximum absolute percentage deviation, and **diff_lin_(x–y),** quantifying percentage differences between measured and actual distances for specific point pairs (Tables 6 and 7).

Performance remained consistent across both phases for PIQT and SCALING_T tests, with **nema_perc_dif.** maintaining A + Ppk values and OOC rates for specific point pairs below 2.5%, indicating strong system stability. However, SCALING_C exhibited significant variability in Phase I, with OOS events leading to a Ppk at the C level. These issues were resolved in Phase II, improving Ppk to A +, though the maximum OOC rate for specific point pairs rose to 21.2% due to baseline shifts following routine maintenance between the 197th and 198th measurement points. This maintenance notably improved spatial linearity, reducing mean deviations for **diff_lin_(2-44)** from -0.08% to -0.02%, **diff_lin_(3-43)** from -0.11% to -0.06%, and **diff_lin_(4-42)** from -0.21% to -0.16%, triggering OOC events despite improved performance. These trends align with changes observed in **nema_perc_dif.** (Fig. 4e).

**Fig. 5:**
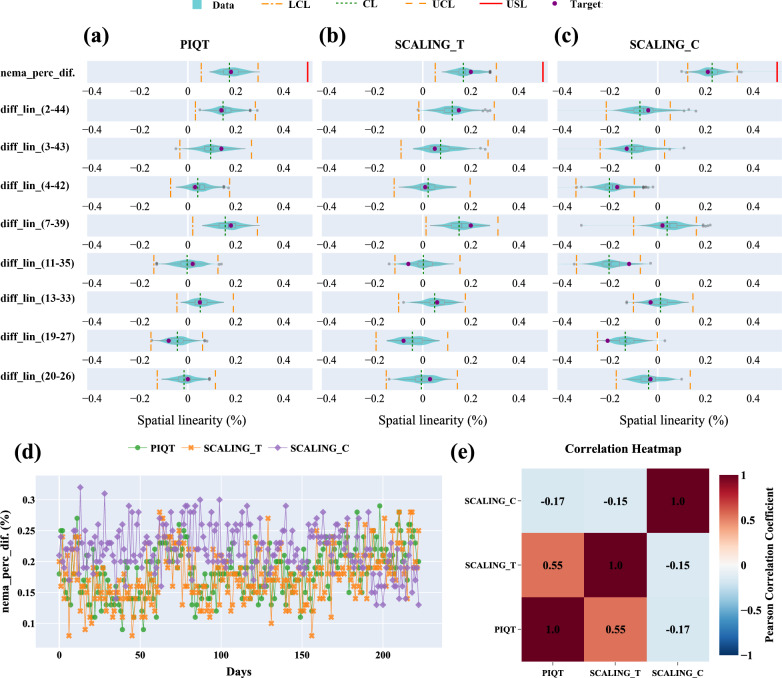
Spatial linearity assessment across PIQT (**a**), SCALING_T (**b**), and SCALING_C (**c**), showing **nema_perc_dif.** and **diff_lin_(x–y)** metrics for specific point pairs. Violin plots illustrate the distribution of spatial linearity deviations, overlaid with central lines (CL, green dotted lines), control limits (UCL, LCL, orange dashed and dash-dot lines), and specification limits (LSL, USL, red dashed lines). Purple points represent target or baseline values. **d** Temporal variation of **nema_perc_dif**. across the three tests, excluding OOC data. **e** Correlation heatmap showing inter-test relationships after excluding OOC data

Figure 4 compare spatial linearity metrics across PIQT, SCALING_T, and SCALING_C tests. PIQT and SCALING_T demonstrated strong stability, with metrics clustering tightly near the central line (CL) and well within control limits (UCL, LCL) (Fig. 4a, b), as further supported by the positive correlation between these tests (r = 0.55; Fig. 5e). In contrast, SCALING_C exhibited higher variability, with **diff_lin_(x–y)** values for pairs like 2–44 and 7–39 approaching or exceeding control limits (Fig. 5c). Temporal trends of **nema_perc_dif.** in SCALING_C showed wider dispersion (Fig. 5d) and weaker correlations with PIQT (r = − 0.17) and SCALING_T (r = − 0.15) (Fig. 5e). These findings indicate weaker spatial linearity consistency between the horizontal and transverse planes, emphasizing the need for simultaneous measurements to ensure comprehensive evaluation.

#### Slice profile

The slice profile was assessed using **Nema_Slice_int.**, **Nema_FWHM**, and **Nema_FWTM**, representing slice integral, full width at half maximum, and full width at tenth maximum, respectively. **Nema_Slice_int.** and **Nema_FWHM** demonstrated consistently stable performance across both phases, with Ppk values above 1.33 and no OOS points, indicating well-controlled slice integrity and sharpness (Table 7). In contrast, **Nema_FWTM** exhibited higher variability in Phase I, particularly for **QA1H:MS,SE (30)**, which uses Marlin anterior/posterior coils with spin-echo sequences and a 30 ms echo time, where Ppk dropped to 1.08 with two OOS events. In Phase II, Ppk increased significantly to 3.22 with no OOS points, reflecting improved slice resolution and stabilized processes. Overall, **Nema_Slice_int.** and **Nema_FWHM** remained stable, while **Nema_FWTM** showed marked improvement, emphasizing the enhanced control of slice profile performance.

#### Spatial resolution

Spatial resolution was assessed using **Ver_pxl_size** (vertical pixel size) and **Hor_pxl_size** (horizontal pixel size). Both parameters maintained process stability across Phase I and Phase II, with no OOC or OOS events observed (Fig. 4g, h). However, **Ver_pxl_size** showed consistently lower performance, with Ppk values ranging from 1.04 to 1.10 (B-grade), highlighting opportunities for optimization. In contrast, **Hor_pxl_size** demonstrated superior performance, with Ppk values ranging from 1.76 to 2.19, indicating greater consistency and stronger control of spatial resolution.

### 3D Geometric distortion

Geometric distortion was evaluated within spherical volumes of varying diameters (DSVs: 200 mm, 300 mm, 400 mm, and 500 mm), excluding 2% of markers with the largest distortions, as illustrated in Fig. A3 in Additional File 1. Distortion was assessed across the AP (anterior–posterior), RL (right-left), and FH (foot-head) directions, with maximum deviation calculated for each DSV. Across both phases, no OOS points were observed, and Ppk values consistently exceeded 1.67, indicating high geometric accuracy (Table 8). However, Phase II exhibited a slight increase in OOC points, primarily in smaller DSVs and the AP direction.

**Table 8:**
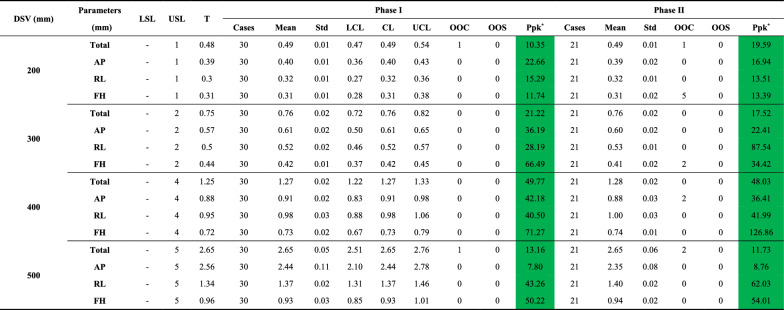
Summary of DSV geometric accuracy metrics across different directions in Phase I and Phase II

DSV = diameter of spherical volume; AP = anterior–posterior; RL = right-left; FH = foot-head; LSL = lower specification limit; USL = upper specification limit; T = baseline (target) value; Std = standard deviation; LCL = lower control limit; CL = central line; UCL = upper control limit; OOC = out-of-control cases; OOS = out-of-specification cases; Ppk = process performance index; –: not provided

*: Color coding for Ppk: Green: A + (Ppk ≥ 1.67); Light green: A (1.67 > Ppk ≥ 1.33); Yellow: B (1.33 > Ppk ≥ 1.00); Light red: C (Ppk < 1.00)

**Fig. 6:**
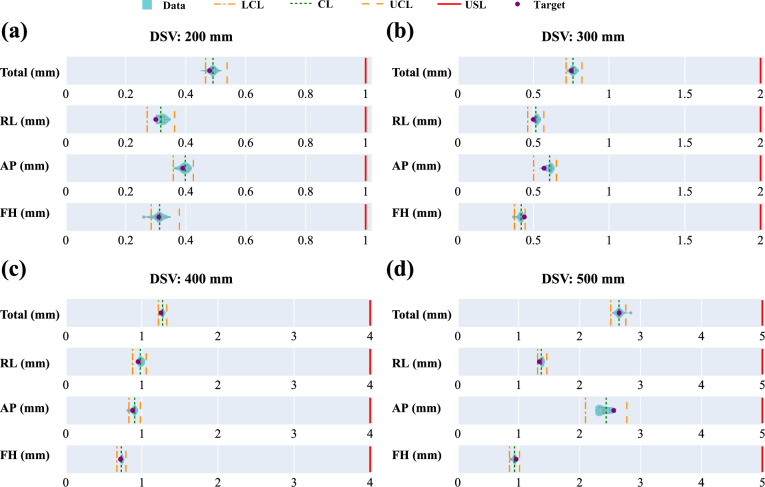
Geometric accuracy for DSVs (diameter of spherical volumes) across sphere diameters: **a** 200 mm, **b** 300 mm, **c** 400 mm, and **d** 500 mm. Violin plots illustrate the distribution of deviations for total and individual directions (RL = right-left, AP = anterior–posterior, FH = foot-head), overlaid with central lines (CL, green dotted lines), control limits (UCL, LCL, orange dashed and dash-dot lines), and specification limits (LSL, USL, red dashed lines). Purple points represent target values

Distortion magnitude increased with DSV size, rising from ~ 0.49 mm at 200 mm to 2.65 mm at 500 mm (Fig. 6). Directional analysis revealed the AP direction consistently exhibited the highest distortion, with a maximum of 2.44 mm at 500 mm, compared to 1.37 mm in the RL direction and 0.93 mm in the FH direction. In contrast, the FH direction demonstrated the lowest distortion across all DSVs, reflecting stable control along this axis.

At 400 mm DSV, mean distortions across all directions remained within 1 mm, reflecting well-controlled geometric accuracy. For smaller DSVs (200 mm and 300 mm), distortions were more uniform across directions, with minimal differences among the AP, RL, and FH axes. These findings comprehensively characterize geometric distortion trends, illustrating the relationship between DSV size, directional variability, and overall geometric accuracy.

## Discussion

This study provides a detailed evaluation of the Elekta Unity MR-Linac’s performance stability over a 1-year clinical operation period using statistical process control (SPC) techniques. By analyzing QA metrics across beam quality, MR-to-MV alignment, MR image quality, and 3D geometric distortion, the study highlights the system’s performance to maintain high precision while identifying critical areas for improvement to support ART workflows. Unlike previous studies that reported longitudinal QA trends for Elekta Unity MR-Linacs [18, 20], the use of SPC enables a more reliable and quantitative analysis of performance variability, offering actionable insights.

Daily QA of beam quality is essential for ensuring treatment accuracy [30]. The inclusion of gantry 180° testing in this study accounts for structural differences between MR-Linacs and conventional Linacs, allowing for the detection of dose variations caused by factors such as water accumulation or foreign objects within the aperture. While beam quality metrics showed consistent trends across gantry angles, the additional testing at 180° proved valuable for identifying potential issues that might otherwise go undetected (Fig. 1).

SPC analysis revealed varying stability across beam quality metrics. Parameters such as **AxSym**, **Energy**, **YSize**, and **YShift** demonstrated strong performance across both gantry angles, with Ppk values exceeding 1.33 and OOC rates below 5.0%. However, metrics like **Dose**, **TrSym**, **XSize**, and **XShift** exhibited significantly higher variability in Phase II. For instance, the Ppk for **Dose** at gantry 0° dropped from 3.13 in Phase I to 1.33 in Phase II, with the OOC rate increasing to 43.6%, likely due to baseline shifts following recalibration of the output dose. Variability in **TrSym**, **XSize**, and **XShift** suggests other factors, such as random positioning errors or operational drift, may also contribute. The concurrent increases in variability among **XShift**, **TrSym**, and **XSize** during Phase II indicate potential interdependencies that warrant further investigation to enhance beam quality consistency. Field size and field shift measurements, as designed for light-field coincidence testing [30], faced limitations when adapted to the Daily QA-MR device. During alignment, the device relied on a ceiling-mounted sagittal laser and an index bar for cranial-caudal positioning, introducing higher susceptibility to random errors and laser drift along the X-axis. This likely contributed to the lower stability observed in field size, field shift, and symmetry along the X-axis compared to the Y-axis. These findings emphasize the importance of refining alignment protocols and minimizing positioning uncertainties to enhance QA reliability.

The MR-to-MV alignment test is critical for accurate online ART planning, as the treatment planning system (TPS) depends on this relationship [3]. SPC-based analysis demonstrated strong overall performance, with translational and rotational offsets maintaining A + levels (Ppk ≥ 1.80) across most metrics. However, Phase II revealed declines in the Phi rotational offset (Ppk = 1.21) and the Z translational offset (Ppk = 1.80). Increased variability in the Z translational offset was first detected during Phase I (Fig. 2c), coinciding with an MR system upgrade from R6.0.5331 to R6.1.571, which involved replacing the system hard drive. While post-upgrade tests, including PIQT, showed no abnormalities, SPC identified subtle performance declines, demonstrating its sensitivity to changes often undetected by routine QA. An OOS event in the Z rotational offset during Phase II was effectively resolved by restarting the accelerator and Marlin MR system, restoring all parameters to normal. This highlights the importance of regular system checks and real-time SPC monitoring to proactively address performance deviations. In contrast, the Phi rotational offset showed significant deterioration in Phase II, with an 86% OOC rate, emphasizing the vulnerability of rotational alignment to long-term drift. While these variations remained within specification limits, understanding their root causes could provide valuable insights into system performance dynamics. These findings underscore the value of integrating SPC into routine QA workflows to detect, quantify, and address both gradual and abrupt performance deviations, ensuring long-term system reliability.

The MR image quality assessments in Phase II showed notable improvements, with no OOS events and enhanced stability across key metrics. FFU metrics, including SNR and uniformity, consistently achieved A + level Ppk values, reflecting stable system performance. Corrective actions in Phase I resolved operational errors, enabling full recovery in Phase II. Spatial linearity in the transverse planes (SCALING_T and PIQT) remained well-controlled across both phases with A + level Ppk values. In contrast, coronal plane linearity (SCALING_C), despite significant improvements after resolving Phase I OOS events, exhibited increased OOC rates (21.2%) in Phase II due to baseline shifts following routine maintenance, as indicated by altered trends in **diff_lin_(2-44)** and **diff_lin_(3-43)**. This highlights the sensitivity of coronal linearity to systematic adjustments. Slice profile metrics, such as **Nema_Slice_int.** and **Nema_FWHM**, demonstrated stable performance, while Phase I variability in **Nema_FWTM** was resolved in Phase II, achieving a Ppk of 3.22, indicative of improved slice resolution and control. Spatial resolution metrics remained stable overall, but the persistent lower Ppk values (B-grade) of vertical pixel size indicate the need for targeted improvements in this parameter. These findings demonstrate the utility of SPC in detecting subtle trends, quantifying variability, and guiding corrective actions, ensuring consistent imaging quality crucial for the precision demands of ART workflows.

Geometric distortion was well-controlled across both phases, with no OOS events and Ppk values consistently exceeding 1.67 for all DSVs. Distortion increased with DSV size, ranging from ~ 0.49 mm at 200 mm to ~ 2.65 mm at 500 mm, aligning with previous reports [20]. The AP axis consistently showed the highest distortion, with deviations of 2.44 mm at 500 mm compared to 1.37 mm for RL and 0.93 mm for FH, aligning with previous reports [14]. At 400 mm DSV, mean distortions in all directions remained within 1 mm, likely reflecting improved field uniformity and calibration. These results highlight the need to account for geometric distortion when defining planning target volumes (PTVs). For centrally located targets near 400 mm DSV, tighter PTV margins may suffice, whereas larger margins should be applied for peripheral or larger-volume targets to mitigate distortion along the AP axis and ensure accurate dose delivery. Routine QA protocols should incorporate these considerations to enhance precision in ART planning.

The primary strength of this study lies in the systematic use of SPC techniques, integrating control charts and process performance analysis to dynamically monitor MR-Linac performance, providing a comprehensive framework for identifying stable parameters and areas for optimization. However, the exclusion of monthly and annual QA tests limits the ability to capture long-term trends or rare anomalies. Additionally, the lighter clinical workload of the Unity system (averaging 10–15 cases per day) may affect the generalizability of the findings. Reduced PIQT testing frequency during certain periods (2–3 times per week) further limits the comprehensiveness of the analysis. In the context of long-term QA monitoring, recalculation of control limits should be approached judiciously. Major process changes, such as hardware upgrades, system recalibrations, or persistent baseline shifts, warrant the establishment of new control limits based on post-change data. If process degradation persists and stabilizes at a suboptimal level (e.g., due to equipment aging), recalibration may be necessary to maintain efficient monitoring. However, consistently low process performance (e.g., Ppk < 1.00) despite stability may indicate the need for equipment replacement or system upgrades. Future studies should validate these findings across multiple institutions and systems to enhance broader applicability. Integrating machine learning with SPC techniques could enable earlier detection of deviations, while efforts to address geometric distortion, particularly in larger DSVs and along the AP axis, are crucial for optimizing clinical workflows and improving treatment precision.

Moreover, while this study primarily focused on I-Charts due to their simplicity and suitability for routine QA, the use of more agile SPC methods, such as cumulative sum (CUSUM) control charts [31, 32] and exponentially weighted moving average (EWMA) charts [33, 34], may offer additional advantages for earlier detection of minor process shifts, particularly in high-frequency or real-time QA monitoring environments. Exploring such methodologies could further strengthen adaptive quality assurance strategies in future work.

## Conclusions

This study systematically evaluated the long-term performance of the Elekta Unity MR-Linac using SPC techniques, providing dynamic insights into beam quality, MR-to-MV alignment, imaging quality, and geometric distortion. While most parameters exhibited acceptable stability and compliance with specifications, key areas for improvement were identified, including beam quality variability, MR-to-MV rotational misalignment, vertical spatial resolution, and geometric distortion along the AP axis in larger DSVs. These findings underscore the clinical importance of integrating advanced QA methodologies like SPC to detect subtle trends, implement timely corrective actions, and ensure optimal system performance. Future work should prioritize multi-center validation and targeted strategies to address these variability factors, further enhancing precision in adaptive radiotherapy.

## Supplementary Information

Supplementary material 1 (DOCX 9350 KB)

Supplementary material 2 (DOCX 247 KB)

## Data Availability

The datasets used and/or analyzed during the current study are available from the corresponding author on reasonable request.

## Abbreviations

AxSym: Axial symmetry
ART: Adaptive radiotherapy
AP: Anterior-posterior
CL: Central line
diff_lin(x–y): Differential linearity between points x and y
DSV: Diameter of spherical volume
FOV: Field of view
FFU: Flood field uniformity
Hor_pxl_size: Horizontal pixel size
LCL: Lower control limit
Linac: Linear accelerator
LSL: Lower specification limit
MR: Magnetic resonance
MR-Linac: Magnetic resonance linear accelerator
MV: Megavoltage
MRL_ACPC_T: Scans using all or specific elements of the marlin anterior/posterior coil, e.g., T_1, anterior coil element 1; T_5, posterior coil element 1
NEMA: National Electrical Manufacturers Association
NEMA_FWHM: Slice profile full width at half maximum
NEMA_FWTM: Slice profile full width at tenth maximum
NEMA_Int_Unif: Integral uniformity
NEMA_Slice_int: Integral of slice profile
nema_perc_dif: Maximum absolute percentage deviation defined by NEMA
OOC: Out-of-control
OOS: Out-of-specification
Ppk: Process performance index
PIQT: Periodic image quality test
QA: Quality assurance
RL: Right-left
SNR: Signal-to-noise ratio
SPL: Spatial linearity
SPR: Spatial resolution
SLP: Slice profile
Std: Standard deviation
T: Baseline, target value
TrSym: Transverse symmetry
UCL: Upper control limit
USL: Upper specification limit
Ver_pxl_size: Vertical pixel size
WSD: Weighted standard deviation
XSize: Beam size along the X axis
XShift: Beam shift along the X axis
YSize: Beam size along the Y axis
YShift: Beam shift along the Y axis
