# Supplementary material for: Long-term performance evaluation of a 1.5T MR-Linac using statistical process control techniques
Source: Radiat Oncol. 2025 Jun 7;20:98. doi: 10.1186/s13014-025-02670-3 (PMC12145638; doi:10.1186/s13014-025-02670-3)
Supplement: Supplementary file 1 — Supplementary material 1 (DOCX 9350 KB) [file 13014_2025_2670_MOESM1_ESM.docx]

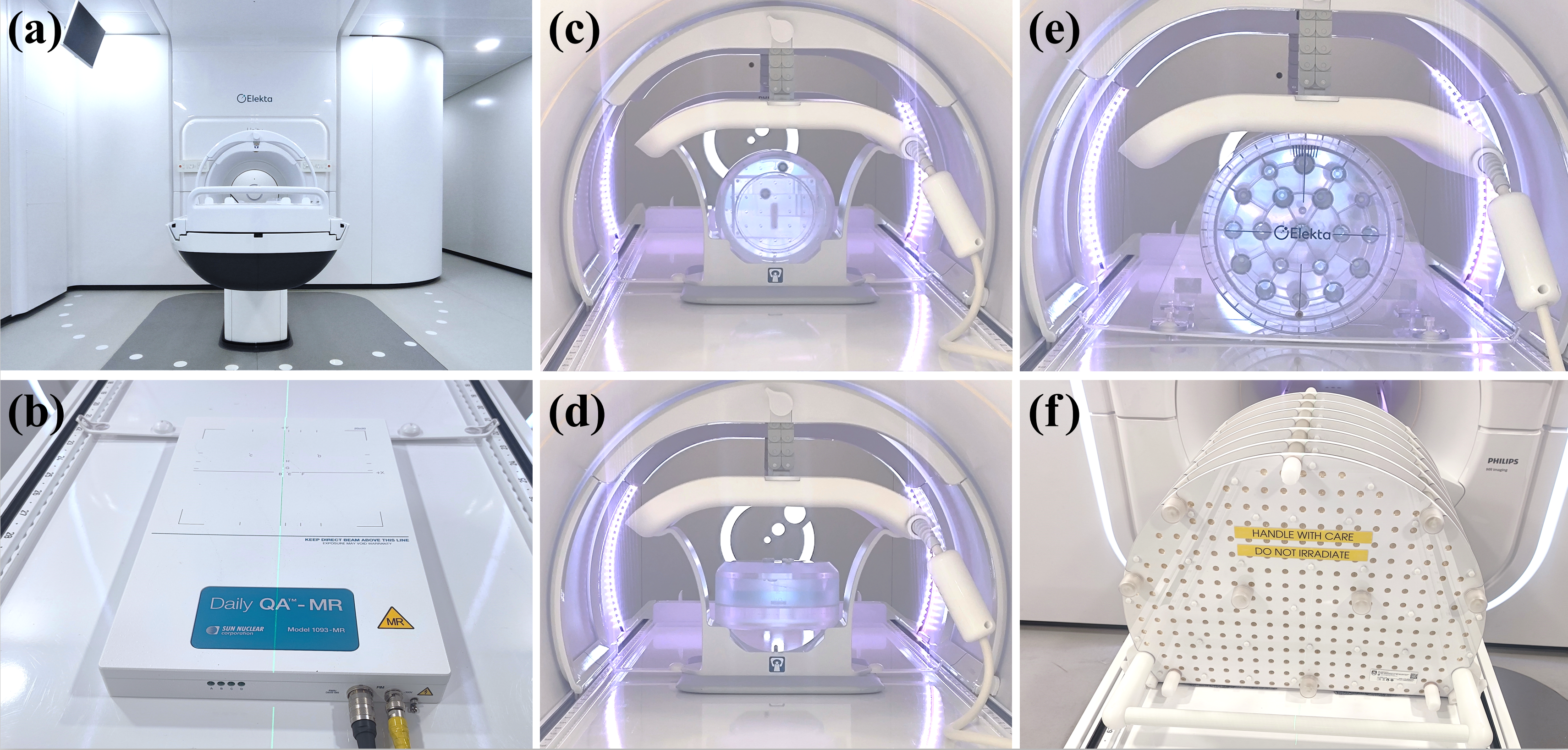


**Figure A1. Quality assurance (QA) setups and tests for the Elekta Unity MR-Linac.**(a) Overview of the Elekta Unity MR-Linac system.
(b) Positioning of the Daily QA-MR phantom for beam quality checks.
(c) Vertical placement of the 200 mm head phantom for the **PIQT**, **SNR Test**, and **MRL_SCALING_T** assessments.
(d) Horizontal placement of the 200 mm head phantom for the **MRL_SCALING_C** test.
(e) Setup for MR-to-MV isocenter alignment testing using the Elekta MR-to-MV QA phantom.
(f) 3D MR geometric distortion test setup with a fiducial grid phantom.


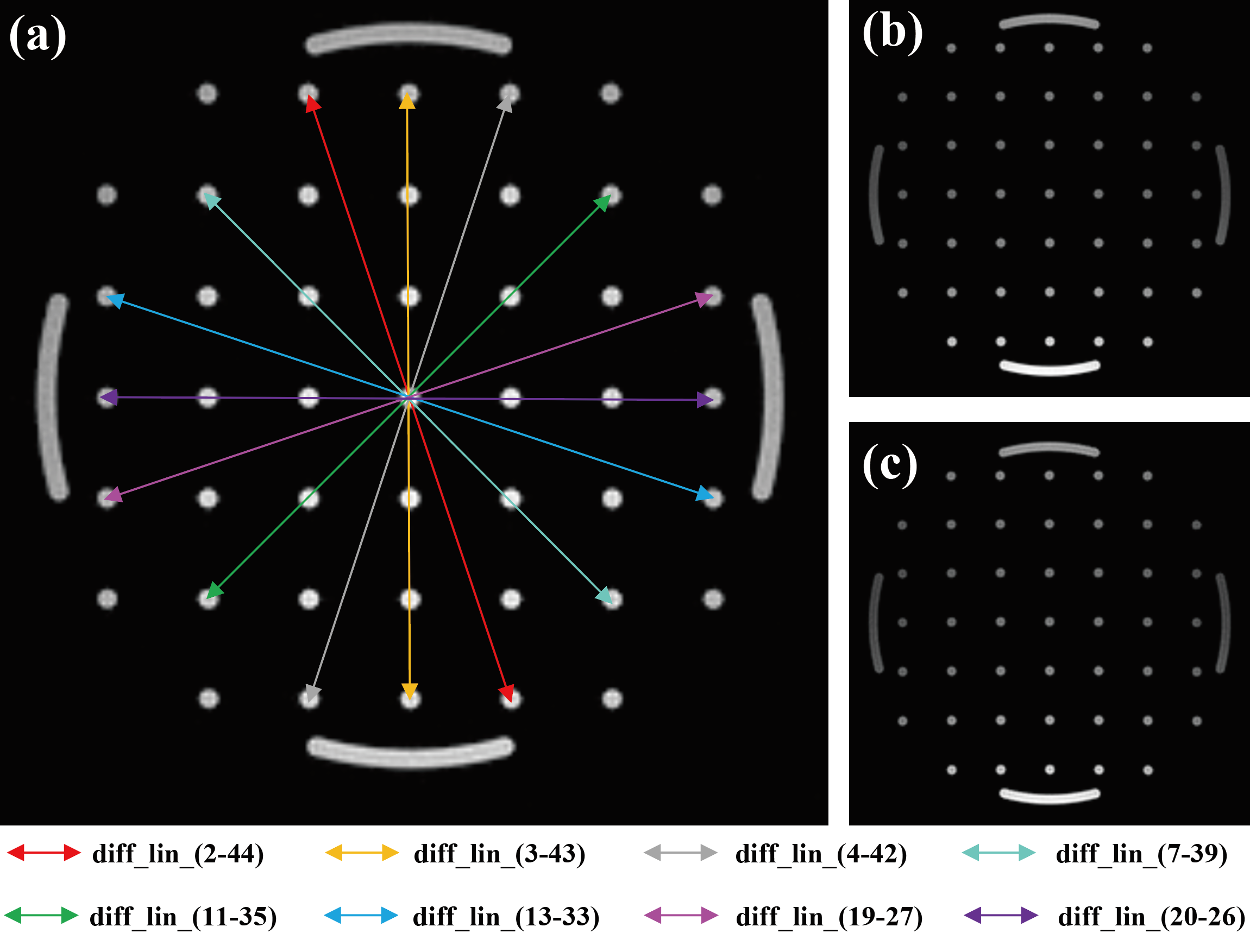


**Figure A2. Geometric accuracy test images using the 200 mm head phantom.**
(a) **MRL_SCALING_C test**: Coronal geometric scaling test, illustrating deviations between paired points (diff_lin_(x-y)) to evaluate geometric scaling accuracy.
(b) **MRL_SCALING_T test**: Transverse geometric scaling test, assessing geometric accuracy in the transverse plane.
(c) **PIQT transverse test**: Transverse geometric scaling evaluation from the periodic image quality test (PIQT), conducted with a spin-echo sequence (TE = 30 ms) using Marlin anterior and posterior coils.


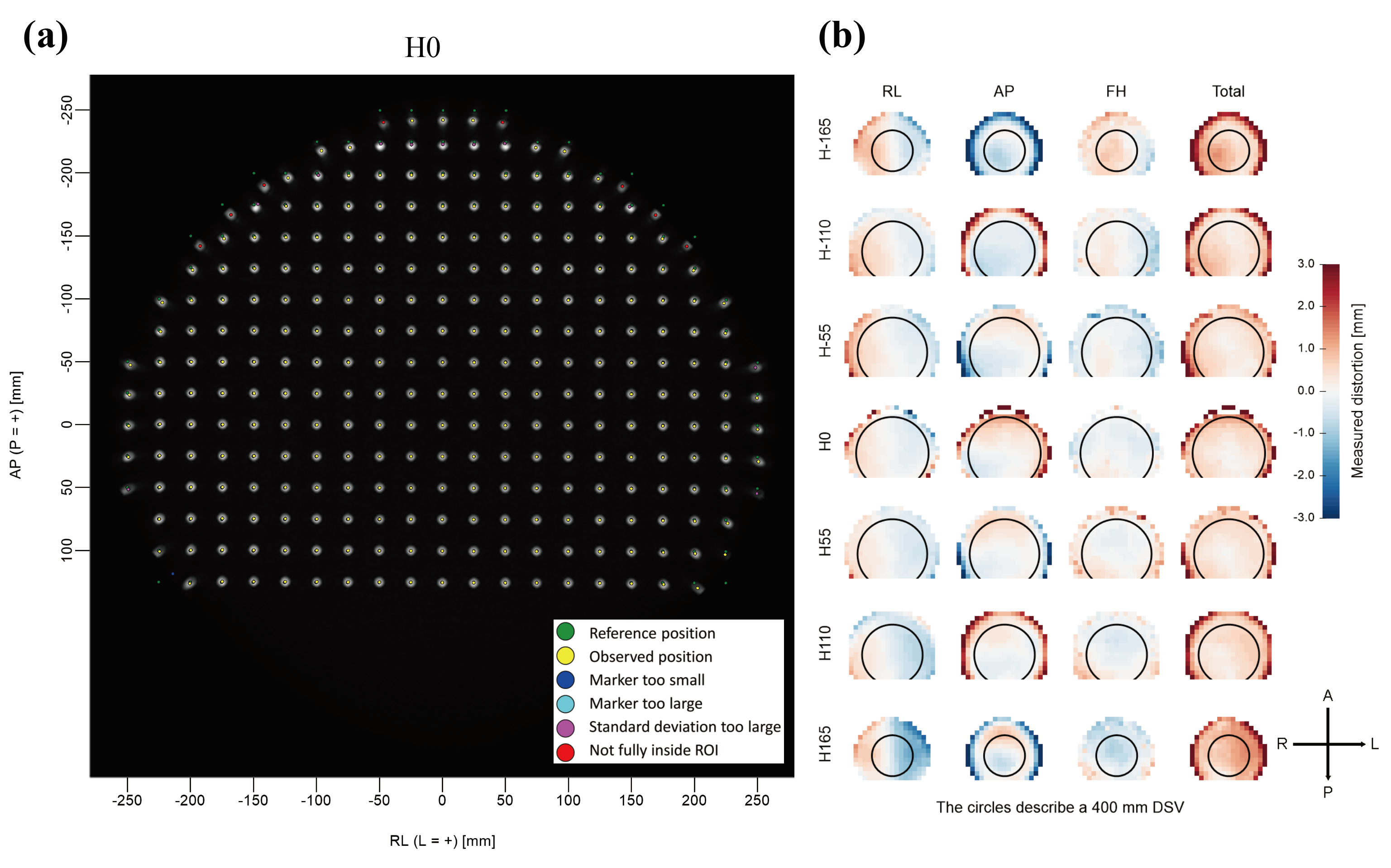


**Figure A3. Results of the 3D geometric distortion test for the Elekta Unity MR-Linac system.**
(a) Representative image of a single slice, with markers color-coded based on analysis outcomes: green for reference positions, yellow for observed positions, blue for markers that are too small, magenta for markers with excessive standard deviation, and red for markers not fully inside the region of interest (ROI).
(b) Distortion maps for a 400 mm diameter spherical volume (DSV), displaying geometric deviations in the right-left (RL), anterior-posterior (AP), and foot-head (FH) directions, as well as the total distortion. Black circles outline the DSV boundary, providing an overview of the Marlin system's geometric accuracy.

**Table A1.** Statistical summary of Skewness, Kurtosis, and Normality tests for DQA tests at Gantry 0° and 180°.

| **DQA Test** | **Parameters** | **PhaseⅠ** | | | | | | **PhaseⅡ** | | | | | |
| --- | --- | --- | --- | --- | --- | --- | --- | --- | --- | --- | --- | --- | --- |
|  |  | **Cases** | **Mean** | **Std** | **AD_p** | **Skewness** | **Kurtosis** | **Cases** | **Mean** | **Std** | **AD_p** | **Skewness** | **Kurtosis** |
| **Gantry 0°** | Dose (cGy) | 150 | 199.19 | 0.58 | 0.000 | 0.05 | 7.65 | 110 | 200.21 | 1.08 | 0.000 | -0.94 | -0.69 |
|  | AxSym (%) | 150 | -0.18 | 0.31 | 0.012 | -0.60 | 0.09 | 110 | -0.29 | 0.31 | 0.020 | -0.61 | 0.42 |
|  | TrSym (%) | 150 | -0.35 | 0.18 | 0.000 | 0.23 | 2.63 | 110 | -0.50 | 0.25 | 0.007 | 0.38 | 1.70 |
|  | Energy (%) | 150 | -0.06 | 0.09 | 0.029 | 0.48 | -0.45 | 110 | -0.18 | 0.07 | **0.356** | -0.08 | 0.58 |
|  | XSize (cm) | 150 | 19.79 | 0.02 | 0.000 | -1.92 | 12.88 | 110 | 19.81 | 0.03 | 0.019 | 0.57 | -0.58 |
|  | XShift (cm) | 150 | 0.21 | 0.03 | 0.000 | -0.88 | 2.40 | 110 | 0.25 | 0.04 | 0.000 | -0.58 | 3.18 |
|  | YSize (cm) | 150 | 19.86 | 0.02 | 0.000 | -1.82 | 10.14 | 110 | 19.85 | 0.01 | 0.000 | 0.53 | 4.58 |
|  | YShift (cm) | 150 | -0.09 | 0.04 | 0.000 | 1.13 | 2.02 | 110 | -0.07 | 0.04 | 0.000 | 0.94 | 1.60 |
| **Gantry 180°** | Dose (cGy) | 150 | 199.08 | 0.82 | **0.718** | -0.09 | -0.31 | 108 | 199.56 | 1.16 | 0.001 | -0.95 | -0.52 |
|  | AxSym (%) | 150 | 3.89 | 0.36 | 0.004 | -0.67 | 0.23 | 108 | 3.77 | 0.38 | 0.006 | -0.69 | 0.65 |
|  | TrSym (%) | 150 | 4.68 | 0.25 | 0.000 | 0.48 | 2.09 | 108 | 4.33 | 0.34 | 0.001 | 0.51 | 2.32 |
|  | Energy (%) | 150 | -0.06 | 0.10 | **0.053** | 0.21 | -0.65 | 108 | -0.23 | 0.06 | **0.479** | 0.22 | -0.38 |
|  | XSize (cm) | 150 | 19.98 | 0.04 | 0.000 | 4.15 | 19.88 | 108 | 19.97 | 0.03 | 0.000 | 6.30 | 50.32 |
|  | XShift (cm) | 150 | -0.09 | 0.04 | 0.000 | -1.58 | 4.29 | 108 | -0.04 | 0.04 | 0.000 | -1.26 | 6.09 |
|  | YSize (cm) | 150 | 19.94 | 0.02 | 0.000 | 1.13 | 3.18 | 108 | 19.93 | 0.02 | 0.000 | 1.04 | 0.96 |
|  | YShift (cm) | 150 | -0.04 | 0.03 | 0.005 | 0.56 | 0.78 | 108 | -0.03 | 0.03 | **0.067** | 0.42 | 0.63 |

*Abbreviations*: DQA = daily QA; Std = standard deviation; AxSym = axial symmetry, TrSym = transverse symmetry; XSize = beam size along the X axis; XShift = beam shift along the X axis; YSize = beam size along the Y axis; YShift = beam shift along the Y axis; AD_p = *p*-value of the Anderson-Darling normality test.

**Table A2.** Statistical summary of Skewness, Kurtosis, and Normality tests for MR-to-MV alignment performance test.

| **MR to MV** | **Parameters** | **PhaseⅠ** | | | | | | **PhaseⅡ** | | | | | |
| --- | --- | --- | --- | --- | --- | --- | --- | --- | --- | --- | --- | --- | --- |
|  |  | **Cases** | **Mean** | **Std** | **AD_p** | **Skewness** | **Kurtosis** | **Cases** | **Mean** | **Std** | **AD_p** | **Skewness** | **Kurtosis** |
| Rotation  (°) | Psi | 150 | -0.07 | 0.01 | 0.000 | 2.11 | 13.69 | 116 | -0.08 | 0.01 | **0.551** | -0.10 | -0.44 |
|  | Phi | 150 | -0.12 | 0.01 | 0.000 | -0.92 | 0.31 | 116 | -0.09 | 0.04 | 0.000 | -1.37 | -0.05 |
|  | Theta | 150 | -0.09 | 0.00 | **0.309** | 0.26 | -0.33 | 116 | -0.09 | 0.00 | **0.085** | -0.42 | 0.47 |
| Translation  (mm) | X | 150 | -0.57 | 0.02 | **0.170** | 0.31 | 0.33 | 116 | -0.60 | 0.01 | **0.458** | -0.28 | -0.11 |
|  | Y | 150 | 3.15 | 0.06 | 0.009 | 0.62 | -0.29 | 116 | 3.14 | 0.05 | **0.793** | 0.14 | -0.19 |
|  | Z | 150 | 0.17 | 0.09 | 0.000 | 1.22 | 0.57 | 116 | 0.27 | 0.18 | 0.000 | 2.01 | 8.31 |

*Abbreviations*: MR to MV: Magnetic resonance to megavoltage alignment; Std = standard deviation; AD_p = *p*-value of the Anderson-Darling normality test.

**Table A3.** Statistical summary of Skewness, Kurtosis, and Normality tests for MR image quality tests.

| **Category** | **Image  Metric** | **Scan_Name** | **Parameters** | **Unit** | **PhaseⅠ** | | | | | | **PhaseⅡ** | | | | | |
| --- | --- | --- | --- | --- | --- | --- | --- | --- | --- | --- | --- | --- | --- | --- | --- | --- |
|  |  |  |  |  | **Cases** | **Mean** | **Std** | **AD_p** | **Skewness** | **Kurtosis** | **Cases** | **Mean** | **Std** | **AD_p** | **Skewness** | **Kurtosis** |
| **MRL_QA_ SNR** | **Flood Field  Uniformity** | MRL_ACPC_T | Nema S/N (B) | None | 150 | 124.71 | 5.30 | 0.000 | -1.01 | 4.19 | 80 | 124.99 | 4.30 | **0.854** | -0.03 | 0.10 |
|  |  | MRL_ACPC_T_1 |  |  | 150 | 108.74 | 8.22 | 0.000 | -5.87 | 42.25 | 80 | 111.17 | 4.40 | 0.000 | 0.89 | 1.87 |
|  |  | MRL_ACPC_T_2 |  |  | 150 | 146.57 | 11.46 | 0.000 | -6.11 | 44.49 | 80 | 149.00 | 5.37 | **0.201** | 0.34 | 0.55 |
|  |  | MRL_ACPC_T_3 |  |  | 150 | 160.99 | 12.57 | 0.000 | -5.93 | 42.80 | 80 | 165.54 | 6.23 | **0.616** | 0.25 | -0.25 |
|  |  | MRL_ACPC_T_4 |  |  | 150 | 102.17 | 8.13 | 0.000 | -4.79 | 32.11 | 80 | 104.60 | 4.41 | **0.485** | -0.01 | -0.59 |
|  |  | MRL_ACPC_T_5 |  |  | 150 | 114.93 | 4.55 | **0.086** | 0.42 | 0.20 | 80 | 116.13 | 4.96 | **0.320** | -0.10 | 0.70 |
|  |  | MRL_ACPC_T_6 |  |  | 150 | 246.63 | 8.62 | **0.419** | 0.25 | -0.21 | 80 | 248.09 | 10.38 | 0.007 | -0.75 | 0.86 |
|  |  | MRL_ACPC_T_7 |  |  | 150 | 243.33 | 9.12 | **0.750** | 0.13 | 0.02 | 80 | 243.35 | 10.39 | 0.022 | -0.41 | 1.52 |
|  |  | MRL_ACPC_T_8 |  |  | 150 | 118.62 | 5.36 | **0.204** | 0.34 | -0.25 | 80 | 119.47 | 5.42 | 0.014 | -0.54 | 1.37 |
|  |  | MRL_ACPC_T | Nema_Int_Unif | % | 150 | 99.26 | 0.07 | 0.023 | 0.54 | 0.16 | 80 | 99.26 | 0.07 | 0.011 | 0.79 | 0.45 |
|  |  | MRL_ACPC_T_1 |  |  | 150 | 99.95 | 0.00 | NaN | 0.00 | NaN | 80 | 99.95 | 0.00 | NaN | 0.00 | NaN |
|  |  | MRL_ACPC_T_2 |  |  | 150 | 99.95 | 0.00 | NaN | 0.00 | NaN | 80 | 99.95 | 0.00 | NaN | 0.00 | NaN |
|  |  | MRL_ACPC_T_3 |  |  | 150 | 99.95 | 0.00 | NaN | 0.00 | NaN | 80 | 99.95 | 0.00 | NaN | 0.00 | NaN |
|  |  | MRL_ACPC_T_4 |  |  | 150 | 99.95 | 0.00 | NaN | 0.00 | NaN | 80 | 99.95 | 0.00 | NaN | 0.00 | NaN |
|  |  | MRL_ACPC_T_5 |  |  | 150 | 99.95 | 0.00 | NaN | 0.00 | NaN | 80 | 99.95 | 0.00 | NaN | 0.00 | NaN |
|  |  | MRL_ACPC_T_6 |  |  | 150 | 99.95 | 0.00 | NaN | 0.00 | NaN | 80 | 99.95 | 0.00 | NaN | 0.00 | NaN |
|  |  | MRL_ACPC_T_7 |  |  | 150 | 99.95 | 0.00 | NaN | 0.00 | NaN | 80 | 99.95 | 0.00 | NaN | 0.00 | NaN |
|  |  | MRL_ACPC_T_8 |  |  | 150 | 99.95 | 0.00 | NaN | 0.00 | NaN | 80 | 99.95 | 0.00 | NaN | 0.00 | NaN |
| **MRL_QA_ SCALING** | **Spatial  Linearity** | MRL_SCALING_T | nema_perc_dif. | % | 150 | 0.17 | 0.04 | 0.044 | 0.51 | -0.13 | 80 | 0.19 | 0.04 | **0.852** | 0.14 | -0.25 |
|  |  |  | diff_lin_(2-44) |  | 150 | 0.12 | 0.05 | **0.079** | 0.42 | 0.24 | 80 | 0.13 | 0.04 | **0.472** | 0.02 | 0.52 |
|  |  |  | diff_lin_(3-43) |  | 150 | 0.07 | 0.06 | **0.055** | 0.49 | -0.05 | 80 | 0.07 | 0.05 | **0.604** | 0.25 | -0.30 |
|  |  |  | diff_lin_(4-42) |  | 150 | 0.02 | 0.05 | **0.472** | 0.17 | -0.36 | 80 | 0.03 | 0.04 | **0.539** | 0.05 | 0.43 |
|  |  |  | diff_lin_(7-39) |  | 150 | 0.15 | 0.05 | **0.720** | 0.15 | -0.16 | 80 | 0.19 | 0.05 | **0.544** | 0.21 | -0.45 |
|  |  |  | diff_lin_(11-35) |  | 150 | 0.00 | 0.05 | **0.607** | -0.08 | 0.26 | 80 | 0.01 | 0.05 | **0.413** | 0.05 | -0.62 |
|  |  |  | diff_lin_(13-33) |  | 150 | 0.05 | 0.05 | **0.513** | -0.12 | -0.39 | 80 | 0.07 | 0.04 | **0.093** | 0.32 | -0.74 |
|  |  |  | diff_lin_(19-27) |  | 150 | -0.04 | 0.05 | 0.003 | 0.08 | -0.83 | 80 | -0.04 | 0.05 | **0.302** | -0.23 | -0.60 |
|  |  |  | diff_lin_(20-26) |  | 150 | -0.01 | 0.05 | **0.073** | -0.25 | -0.59 | 80 | 0.01 | 0.05 | **0.830** | 0.03 | -0.40 |
|  |  | MRL_SCALING_C | nema_perc_dif. | % | 149 | 0.23 | 0.07 | 0.000 | 7.90 | 79.23 | 80 | 0.20 | 0.04 | **0.437** | -0.26 | -0.48 |
|  |  |  | diff_lin_(2-44) |  | 149 | -0.08 | 0.07 | 0.000 | -6.28 | 58.77 | 80 | -0.02 | 0.07 | **0.074** | 0.19 | -0.82 |
|  |  |  | diff_lin_(3-43) |  | 149 | -0.11 | 0.09 | 0.000 | -7.34 | 72.96 | 80 | -0.06 | 0.07 | **0.517** | 0.15 | -0.53 |
|  |  |  | diff_lin_(4-42) |  | 149 | -0.21 | 0.07 | 0.000 | -6.20 | 57.92 | 80 | -0.16 | 0.07 | 0.001 | 0.12 | -1.08 |
|  |  |  | diff_lin_(7-39) |  | 149 | 0.04 | 0.05 | 0.000 | -2.23 | 13.15 | 80 | 0.08 | 0.06 | **0.718** | 0.21 | -0.20 |
|  |  |  | diff_lin_(11-35) |  | 149 | -0.21 | 0.06 | 0.000 | -2.16 | 14.52 | 80 | -0.17 | 0.05 | **0.356** | 0.38 | -0.26 |
|  |  |  | diff_lin_(13-33) |  | 149 | 0.01 | 0.04 | **0.396** | -0.17 | 0.33 | 80 | 0.02 | 0.05 | **0.805** | -0.10 | -0.38 |
|  |  |  | diff_lin_(19-27) |  | 149 | -0.14 | 0.04 | **0.390** | 0.27 | -0.05 | 80 | -0.12 | 0.04 | **0.178** | 0.42 | 0.37 |
|  |  |  | diff_lin_(20-26) |  | 149 | -0.04 | 0.05 | 0.000 | 0.16 | -1.06 | 80 | -0.04 | 0.05 | 0.006 | 0.82 | 0.69 |
| **PIQT** | **Flood Field  Uniformity** | QA1H:MS,SE (100) | Nema S/N (B) | None | 150 | 54.34 | 1.26 | 0.000 | -3.11 | 17.28 | 80 | 54.44 | 0.97 | **0.066** | -0.38 | 1.04 |
|  |  | QA1H:MS,SE (30) |  |  | 150 | 73.68 | 1.83 | 0.000 | -2.15 | 9.67 | 80 | 74.15 | 1.60 | **0.104** | -0.37 | 0.83 |
|  |  | QA2H:MS,FE (15) |  |  | 150 | 63.33 | 1.80 | 0.000 | -3.14 | 17.93 | 80 | 63.76 | 1.31 | 0.000 | -1.28 | 3.43 |
|  |  | QA3B:2D,SE (100) |  |  | 150 | 52.04 | 1.00 | 0.006 | -0.60 | 0.47 | 80 | 51.88 | 1.02 | 0.032 | -0.71 | 0.19 |
|  |  | QA3B:2D,SE (150) |  |  | 150 | 41.59 | 0.83 | 0.000 | -0.81 | 1.67 | 80 | 41.46 | 0.73 | 0.026 | -0.54 | 1.04 |
|  |  | QA3B:2D,SE (50) |  |  | 150 | 60.54 | 1.23 | **0.886** | -0.07 | 0.01 | 80 | 60.36 | 1.33 | **0.374** | 0.03 | -0.64 |
|  |  | QA1H:MS,SE (100) | Nema_Int_Unif | % | 150 | 38.63 | 1.32 | 0.000 | 6.06 | 43.45 | 80 | 38.11 | 0.47 | 0.001 | 0.83 | 1.69 |
|  |  | QA1H:MS,SE (30) |  |  | 150 | 38.36 | 1.30 | 0.000 | 6.88 | 51.61 | 80 | 37.87 | 0.41 | **0.277** | -0.23 | 0.61 |
|  |  | QA2H:MS,FE (15) |  |  | 150 | 39.06 | 1.42 | 0.000 | 6.47 | 47.66 | 80 | 38.56 | 0.47 | **0.769** | -0.17 | -0.29 |
|  |  | QA3B:2D,SE (100) |  |  | 150 | 6.89 | 0.30 | **0.699** | 0.14 | 0.05 | 80 | 6.89 | 0.35 | **0.388** | -0.29 | 0.27 |
|  |  | QA3B:2D,SE (150) |  |  | 150 | 7.23 | 0.36 | **0.218** | 0.32 | -0.29 | 80 | 7.25 | 0.34 | **0.972** | -0.04 | -0.24 |
|  |  | QA3B:2D,SE (50) |  |  | 150 | 6.50 | 0.31 | **0.427** | 0.16 | -0.40 | 80 | 6.48 | 0.33 | **0.143** | 0.15 | 1.07 |
|  | **Spatial  Linearity** | QA1H:MS,SE (30) | nema_perc_dif. | % | 150 | 0.17 | 0.04 | **0.185** | 0.34 | -0.30 | 80 | 0.20 | 0.04 | **0.880** | -0.11 | -0.02 |
|  |  |  | diff_lin_(2-44) |  | 150 | 0.15 | 0.04 | **0.178** | 0.37 | 0.00 | 80 | 0.17 | 0.04 | **0.341** | 0.35 | 0.14 |
|  |  |  | diff_lin_(3-43) |  | 150 | 0.09 | 0.05 | **0.323** | 0.23 | 0.28 | 80 | 0.13 | 0.05 | **0.906** | -0.05 | -0.32 |
|  |  |  | diff_lin_(4-42) |  | 150 | 0.04 | 0.04 | **0.422** | 0.22 | 0.17 | 80 | 0.06 | 0.04 | **0.844** | 0.15 | -0.11 |
|  |  |  | diff_lin_(7-39) |  | 150 | 0.16 | 0.05 | **0.355** | 0.25 | -0.30 | 80 | 0.19 | 0.04 | **0.889** | -0.12 | -0.09 |
|  |  |  | diff_lin_(11-35) |  | 150 | 0.00 | 0.04 | **0.056** | -0.48 | -0.01 | 80 | 0.01 | 0.05 | **0.342** | 0.38 | 0.00 |
|  |  |  | diff_lin_(13-33) |  | 150 | 0.05 | 0.04 | **0.217** | 0.34 | -0.20 | 80 | 0.07 | 0.03 | **0.910** | 0.07 | -0.30 |
|  |  |  | diff_lin_(19-27) |  | 150 | -0.04 | 0.04 | **0.495** | 0.19 | 0.17 | 80 | -0.03 | 0.04 | **0.605** | -0.01 | 0.37 |
|  |  |  | diff_lin_(20-26) |  | 150 | -0.02 | 0.04 | **0.477** | 0.23 | 0.00 | 80 | 0.00 | 0.04 | **0.173** | -0.42 | 0.39 |
|  | **Slice  Profile** | QA1H:MS,SE (30) | Nema_Slice_int. | mm | 150 | 5.11 | 0.03 | 0.008 | 0.31 | 1.45 | 80 | 5.11 | 0.03 | **0.214** | -0.08 | -0.72 |
|  |  | QA1H:MS,SE (100) |  |  | 150 | 4.90 | 0.04 | 0.000 | 1.12 | 3.97 | 80 | 4.89 | 0.04 | **0.099** | 0.30 | 1.01 |
|  |  | QA2H:MS,FE (15) |  |  | 150 | 5.14 | 0.04 | **0.069** | 0.41 | 0.35 | 80 | 5.13 | 0.04 | **0.385** | -0.36 | -0.22 |
|  |  | QA1H:MS,SE (30) | Nema_FWHM |  | 150 | 4.98 | 0.02 | 0.034 | 0.35 | 0.84 | 80 | 4.98 | 0.02 | **0.517** | -0.11 | -0.55 |
|  |  | QA1H:MS,SE (100) |  |  | 150 | 4.73 | 0.02 | **0.052** | 0.40 | 0.51 | 80 | 4.73 | 0.02 | **0.975** | -0.03 | -0.25 |
|  |  | QA2H:MS,FE (15) |  |  | 150 | 4.99 | 0.03 | **0.780** | 0.08 | -0.28 | 80 | 4.98 | 0.03 | **0.719** | -0.21 | -0.16 |
|  |  | QA1H:MS,SE (30) | Nema_FWTM |  | 150 | 7.19 | 0.19 | 0.000 | 6.38 | 52.61 | 80 | 7.20 | 0.09 | **0.764** | 0.19 | -0.19 |
|  |  | QA1H:MS,SE (100) |  |  | 150 | 7.76 | 5.05 | 0.000 | -0.59 | -1.01 | 80 | 8.92 | 4.18 | 0.003 | -0.93 | 0.63 |
|  |  | QA2H:MS,FE (15) |  |  | 150 | 9.62 | 2.07 | 0.000 | -2.40 | 10.44 | 80 | 9.61 | 1.31 | 0.000 | 1.49 | 3.14 |
|  | **Spatial  Resolution** | QA1H:MS,SE (30) | Ver_pxl_size | mm | 150 | 1.36 | 0.04 | **0.867** | 0.09 | -0.02 | 80 | 1.36 | 0.04 | **0.738** | 0.13 | 0.13 |
|  |  | QA1H:MS,SE (100) |  |  | 150 | 1.36 | 0.05 | **0.984** | -0.02 | -0.14 | 80 | 1.36 | 0.04 | **0.832** | -0.15 | -0.07 |
|  |  | QA1H:MS,SE (30) | Hor_pxl_size |  | 150 | 1.12 | 0.03 | 0.008 | 0.08 | -0.79 | 80 | 1.12 | 0.03 | **0.101** | 0.52 | -0.51 |
|  |  | QA1H:MS,SE (100) |  |  | 150 | 1.10 | 0.03 | **0.106** | -0.26 | -0.54 | 80 | 1.11 | 0.04 | **0.390** | 0.05 | -0.63 |

*Abbreviations:* SNR = signal-to-noise ratio; MRL_ACPC_T (and subelements) = scans using all or specific elements of the Marlin Anterior/Posterior coil (e.g., T_1 = Anterior coil element 1; T_5 = Posterior coil element 1); NEMA_Int_Unif = integral uniformity; Nema_Int_Unif= integral uniformity; Nema_Slice_int = integral of slice profile; FWHM = full-width at half maximum; FWTM = full-width at tenth maximum; nema_perc_dif. = NEMA-defined maximum absolute percentage deviation; diff_lin_(x-y) = pairwise distance deviation; Ver_pxl_size = vertical pixel size; Hor_pxl_size = horizontal pixel size; QA1H:MS, SE = Marlin Anterior and Posterior coils with spin-echo sequences; QA2H:MS, FE = Marlin Anterior and Posterior coils with gradient-echo sequences; QA3B:2D, SE = System Body Coil (QBC) with spin-echo sequences; NaN = not available; Std = standard deviation; AD_p = *p*-value of the Anderson-Darling normality test.

**Table A4.** Statistical summary of Skewness, Kurtosis, and Normality tests for geometric accuracy metrics.

| **DSV (mm)** | **Parameters  (mm)** | **PhaseⅠ** | | | | | | **PhaseⅡ** | | | | | |
| --- | --- | --- | --- | --- | --- | --- | --- | --- | --- | --- | --- | --- | --- |
|  |  | **Cases** | **Mean** | **Std** | **AD_p** | **Skewness** | **Kurtosis** | **Cases** | **Mean** | **Std** | **AD_p** | **Skewness** | **Kurtosis** |
| 200 | Total | 30 | 0.49 | 0.01 | **0.538** | -0.12 | 0.47 | 21 | 0.49 | 0.01 | 0.024 | -1.04 | 1.41 |
|  | AP | 30 | 0.40 | 0.01 | **0.618** | -0.38 | -0.12 | 21 | 0.39 | 0.02 | **0.568** | -0.33 | -0.85 |
|  | RL | 30 | 0.32 | 0.01 | **0.190** | 0.35 | -1.01 | 21 | 0.32 | 0.01 | **0.585** | -0.28 | -0.87 |
|  | FH | 30 | 0.31 | 0.01 | **0.170** | 0.65 | 0.48 | 21 | 0.31 | 0.02 | **0.518** | -0.48 | -0.73 |
| 300 | Total | 30 | 0.76 | 0.02 | **0.786** | -0.26 | -0.19 | 21 | 0.76 | 0.02 | **0.916** | -0.02 | -0.66 |
|  | AP | 30 | 0.61 | 0.02 | **0.157** | -0.69 | -0.82 | 21 | 0.60 | 0.02 | **0.266** | -0.79 | -0.18 |
|  | RL | 30 | 0.52 | 0.02 | **0.729** | 0.31 | -0.20 | 21 | 0.53 | 0.01 | **0.420** | -0.64 | -0.53 |
|  | FH | 30 | 0.42 | 0.01 | **0.354** | -0.21 | -0.93 | 21 | 0.41 | 0.02 | **0.931** | -0.18 | -0.45 |
| 400 | Total | 30 | 1.27 | 0.02 | **0.881** | -0.10 | -0.57 | 21 | 1.28 | 0.02 | **0.673** | 0.38 | -0.66 |
|  | AP | 30 | 0.91 | 0.02 | **0.360** | -0.04 | -0.96 | 21 | 0.88 | 0.03 | **0.161** | -0.41 | -1.19 |
|  | RL | 30 | 0.98 | 0.03 | **0.527** | 0.09 | -0.85 | 21 | 1.00 | 0.03 | **0.120** | 0.15 | -1.28 |
|  | FH | 30 | 0.73 | 0.02 | **0.284** | -0.29 | -0.95 | 21 | 0.74 | 0.01 | **0.909** | -0.21 | -0.44 |
| 500 | Total | 30 | 2.65 | 0.05 | 0.000 | 1.38 | 3.39 | 21 | 2.65 | 0.06 | **0.788** | 0.12 | 0.04 |
|  | AP | 30 | 2.44 | 0.11 | 0.003 | 0.00 | -1.41 | 21 | 2.35 | 0.08 | **0.212** | 0.88 | -0.53 |
|  | RL | 30 | 1.37 | 0.02 | **0.180** | 0.18 | -1.07 | 21 | 1.40 | 0.02 | **0.792** | -0.29 | -0.21 |
|  | FH | 30 | 0.93 | 0.03 | **0.908** | -0.03 | -0.57 | 21 | 0.94 | 0.02 | **0.147** | 0.82 | 0.42 |

*Abbreviations:* DSV = diameter of spherical volume; AP = anterior-posterior; Std = standard deviation; AD_p = *p*-value of the Anderson-Darling normality test.
