# Supplementary material for: Long-term performance evaluation of a 1.5T MR-Linac using statistical process control techniques
Source: Radiat Oncol. 2025 Jun 7;20:98. doi: 10.1186/s13014-025-02670-3 (PMC12145638; doi:10.1186/s13014-025-02670-3)
Supplement: Supplementary file 2 — Supplementary material 2 (DOCX 247 KB) [file 13014_2025_2670_MOESM2_ESM.docx]

**Example of Control Limits Calculation**

**1 Methodology**

The control limits were established based on an individual control chart (I-chart) framework utilizing the weighted standard deviation (WSD)-based method. The key steps are as follows:

**1.1 Data Preparation**

Collect the sequential QA measurements ($X_{1}$, $X_{2}$, ..., $X_{N}$) for the selected parameter.

**1.2 Moving Range Calculation**

Compute the moving range (MRᵢ) between consecutive data points:

$${MR}_{i}=\left| X_{i}-X_{i-1} \right|$$

**1.3 Average Moving Range**Calculate the average moving range ($\bar{MR}$):

$$\bar{MR}=\frac{\sum_{i=2}^{N} {MR}_{i}}{N-1}$$

**1.4 Central Line (CL)**

Determine the process mean (${CL}_{X}$):

$${CL}_{X}=\bar{X}=\frac{\sum_{i=1}^{N} X_{i}}{N}$$

**1.5 Proportion of Positive Deviations (**$P_{X}$**)**Calculate the proportion of data points above the mean:

$P_{X}=\frac{\sum_{i=1}^{N} I\left( \bar{X}-X_{i} \right)}{N}$, $I\left( x \right)=\left\{ \begin{matrix} 1,x\geq0 \\ 0,x<0 \end{matrix} \right.$

**1.6 Determination of** $\boldsymbol{d}_{\boldsymbol{2}}^{\boldsymbol{WSD}}$

The constant $d_{2}^{WSD}$ is a scaling factor that varies as a function of $P_{X}$ when the subgroup size n is 2. The values of $d_{2}^{WSD}$​ used in this study were obtained from **Table A1** of Chang and Bai [1]. For cases where $P_{X}$< 0.5, the constants corresponding to ${1-P}_{X}$​ were used to ensure symmetry in control limit construction. For other intermediate values, linear interpolation between tabulated values was applied.

**Table A1.** The values of the constant $d_{2}^{WSD}$ corresponding to $P_{X}$.

| $P_{X}$ | $d_{2}^{WSD}$ |
| --- | --- |
| 0.50 | 1.128 |
| 0.52 | 1.126 |
| 0.54 | 1.121 |
| 0.56 | 1.112 |
| 0.58 | 1.099 |
| 0.60 | 1.083 |
| 0.62 | 1.063 |
| 0.64 | 1.039 |
| 0.66 | 1.012 |
| 0.68 | 0.982 |
| 0.70 | 0.947 |

**1.7 Control Limits (LCL and UCL)**
Calculate the lower and upper control limits:

| ${LCL}_{X}=\bar{X}-\frac{3\cdot\bar{MR}}{d_{2}^{WSD}}{\cdot2\left( 1-P \right.}_{X})$ |
| --- |
| ${UCL}_{X}=\bar{X}+\frac{3\cdot\bar{MR}}{d_{2}^{WSD}}{\cdot2P}_{X}$ |

**1.8 Phase I Implementation**

(1) For each evaluation metric, one random sample was drawn from the original Phase I dataset, preserving the original sample size and distribution characteristics.

(2) Control limits—including the lower control limit (LCL), central line (CL), and upper control limit (UCL)—were initially calculated using the WSD-based method.

(3) Data points outside the calculated control limits were identified as out-of-control (OOC) and removed.

(4) Using the remaining in-control data, the control limits were recalculated.

(5) Steps (3) and (4) were repeated iteratively until all data points fell within control limits and the dataset achieved statistical control.

(6) The final set of control limits from this refined dataset was recorded as LCL₁, CL₁, and UCL₁.

(7) Steps (1) through (6) were repeated 1,000 times independently, resulting in 1,000 sets of control limits.

(8) The **median values** of LCLⱼ, CLⱼ, and UCLⱼ (j = 1 to 1,000) were then adopted as the final control limits (i.e., tolerance limits) for the corresponding QA metric.

**2 Worked-out Example for Control Limits Calculation**

**2.1 Example Metric**

A total of 150 daily output dose measurements at gantry 0° were collected during Phase I (**Table A2**).

**Table A2.** Dataset of daily output dose measurements at gantry 0° used for the control limit calculation example.

| Measure-  Ment No. | Dose (cGy) | Measure-  Ment No. | Dose (cGy) | Measure-  Ment No. | Dose (cGy) | Measure-  Ment No. | Dose (cGy) | Measure-  Ment No. | Dose (cGy) |
| --- | --- | --- | --- | --- | --- | --- | --- | --- | --- |
| 1 | 199.56 | 31 | 199.72 | 61 | 198.60 | 91 | 199.05 | 121 | 198.68 |
| 2 | 199.69 | 32 | 199.39 | 62 | 199.21 | 92 | 198.96 | 122 | 198.99 |
| 3 | 199.67 | 33 | 199.81 | 63 | 198.69 | 93 | 198.94 | 123 | 198.98 |
| 4 | 199.98 | 34 | 199.83 | 64 | 198.94 | 94 | 198.83 | 124 | 198.93 |
| 5 | 199.87 | 35 | 199.23 | 65 | 198.62 | 95 | 199.42 | 125 | 198.97 |
| 6 | 199.92 | 36 | 199.44 | 66 | 198.85 | 96 | 198.87 | 126 | 198.46 |
| 7 | 199.46 | 37 | 199.39 | 67 | 198.96 | 97 | 198.77 | 127 | 198.78 |
| 8 | 199.55 | 38 | 199.48 | 68 | 198.91 | 98 | 198.75 | 128 | 199.07 |
| 9 | 200.12 | 39 | 199.12 | 69 | 199.18 | 99 | 198.74 | 129 | 198.92 |
| 10 | 199.67 | 40 | 199.86 | 70 | 199.02 | 100 | 199.05 | 130 | 198.77 |
| 11 | 199.82 | 41 | 200.14 | 71 | 198.88 | 101 | 198.89 | 131 | 198.37 |
| 12 | 199.85 | 42 | 200.27 | 72 | 199.35 | 102 | 199.00 | 132 | 198.69 |
| 13 | 199.55 | 43 | 200.37 | 73 | 199.44 | 103 | 198.60 | 133 | 198.93 |
| 14 | 202.14 | 44 | 199.39 | 74 | 199.70 | 104 | 198.86 | 134 | 198.82 |
| 15 | 199.75 | 45 | 199.19 | 75 | 199.49 | 105 | 199.38 | 135 | 196.11 |
| 16 | 200.04 | 46 | 199.21 | 76 | 199.24 | 106 | 199.08 | 136 | 198.72 |
| 17 | 199.83 | 47 | 199.44 | 77 | 199.32 | 107 | 198.79 | 137 | 198.52 |
| 18 | 199.84 | 48 | 199.55 | 78 | 199.51 | 108 | 199.11 | 138 | 198.33 |
| 19 | 199.44 | 49 | 199.34 | 79 | 199.92 | 109 | 199.13 | 139 | 198.58 |
| 20 | 199.79 | 50 | 198.32 | 80 | 199.51 | 110 | 199.00 | 140 | 198.60 |
| 21 | 199.72 | 51 | 198.85 | 81 | 199.72 | 111 | 199.24 | 141 | 198.93 |
| 22 | 200.34 | 52 | 198.97 | 82 | 199.61 | 112 | 199.25 | 142 | 198.55 |
| 23 | 200.07 | 53 | 198.65 | 83 | 199.51 | 113 | 199.25 | 143 | 198.69 |
| 24 | 200.01 | 54 | 199.07 | 84 | 199.71 | 114 | 199.27 | 144 | 198.59 |
| 25 | 199.22 | 55 | 199.28 | 85 | 199.41 | 115 | 198.93 | 145 | 199.01 |
| 26 | 199.55 | 56 | 198.91 | 86 | 199.01 | 116 | 199.02 | 146 | 199.15 |
| 27 | 199.32 | 57 | 198.82 | 87 | 199.22 | 117 | 198.72 | 147 | 198.72 |
| 28 | 199.99 | 58 | 198.35 | 88 | 199.14 | 118 | 198.86 | 148 | 198.84 |
| 29 | 199.34 | 59 | 198.34 | 89 | 198.96 | 119 | 198.95 | 149 | 198.42 |
| 30 | 199.40 | 60 | 198.53 | 90 | 198.97 | 120 | 199.28 | 150 | 198.44 |

**2.2 Step-by-Step Calculation Based on One Randomly Sampled Dataset**

**Step 1: Random Sampling of Phase I Data**

One random sample was generated from the original Phase I dataset by random sampling, maintaining a sample size of 150. The corresponding dataset is presented in **Table A3**.

**Table A3**. Example random sample of daily output dose measurements at gantry 0° used for control limit calculation.

| Measure-  Ment No. | Dose (cGy) | Measure-  Ment No. | Dose (cGy) | Measure-  Ment No. | Dose (cGy) | Measure-  Ment No. | Dose (cGy) | Measure-  Ment No. | Dose (cGy) |
| --- | --- | --- | --- | --- | --- | --- | --- | --- | --- |
| 1 | 198.55 | 31 | 198.82 | 61 | 199.72 | 91 | 199.55 | 121 | 198.35 |
| 2 | 199.39 | 32 | 199.67 | 62 | 199.69 | 92 | 198.97 | 122 | 198.72 |
| 3 | 198.52 | 33 | 199.84 | 63 | 199.15 | 93 | 199.42 | 123 | 199.34 |
| 4 | 198.83 | 34 | 198.84 | 64 | 199.82 | 94 | 199.32 | 124 | 199.87 |
| 5 | 198.87 | 35 | 198.77 | 65 | 198.86 | 95 | 199.12 | 125 | 199.01 |
| 6 | 198.72 | 36 | 199.34 | 66 | 200.34 | 96 | 198.65 | 126 | 199.48 |
| 7 | 198.96 | 37 | 199.79 | 67 | 199.44 | 97 | 198.94 | 127 | 202.14 |
| 8 | 199.19 | 38 | 196.11 | 68 | 199.11 | 98 | 200.27 | 128 | 198.68 |
| 9 | 199.92 | 39 | 199.24 | 69 | 199.27 | 99 | 199.25 | 129 | 199.28 |
| 10 | 199.72 | 40 | 199.83 | 70 | 199.40 | 100 | 198.91 | 130 | 199.25 |
| 11 | 199.21 | 41 | 198.69 | 71 | 198.62 | 101 | 198.97 | 131 | 198.88 |
| 12 | 198.69 | 42 | 198.85 | 72 | 199.92 | 102 | 198.37 | 132 | 199.22 |
| 13 | 199.71 | 43 | 199.13 | 73 | 199.18 | 103 | 199.85 | 133 | 199.44 |
| 14 | 199.55 | 44 | 199.02 | 74 | 199.41 | 104 | 200.12 | 134 | 198.93 |
| 15 | 198.86 | 45 | 199.55 | 75 | 198.32 | 105 | 198.60 | 135 | 199.28 |
| 16 | 198.46 | 46 | 198.92 | 76 | 199.61 | 106 | 198.91 | 136 | 199.67 |
| 17 | 199.39 | 47 | 198.44 | 77 | 198.94 | 107 | 198.59 | 137 | 199.07 |
| 18 | 200.14 | 48 | 200.37 | 78 | 198.42 | 108 | 198.72 | 138 | 200.01 |
| 19 | 198.99 | 49 | 198.74 | 79 | 199.22 | 109 | 198.75 | 139 | 198.60 |
| 20 | 199.86 | 50 | 199.24 | 80 | 199.39 | 110 | 199.56 | 140 | 198.82 |
| 21 | 199.55 | 51 | 198.85 | 81 | 199.51 | 111 | 199.99 | 141 | 198.53 |
| 22 | 199.05 | 52 | 199.51 | 82 | 198.95 | 112 | 199.21 | 142 | 198.96 |
| 23 | 199.00 | 53 | 198.89 | 83 | 198.77 | 113 | 198.33 | 143 | 198.34 |
| 24 | 199.83 | 54 | 199.07 | 84 | 199.70 | 114 | 200.07 | 144 | 198.60 |
| 25 | 198.98 | 55 | 199.44 | 85 | 199.81 | 115 | 198.93 | 145 | 199.35 |
| 26 | 199.02 | 56 | 198.58 | 86 | 199.98 | 116 | 198.69 | 146 | 200.04 |
| 27 | 198.97 | 57 | 198.93 | 87 | 199.00 | 117 | 199.14 | 147 | 199.08 |
| 28 | 199.23 | 58 | 198.78 | 88 | 199.38 | 118 | 199.75 | 148 | 198.79 |
| 29 | 198.96 | 59 | 199.72 | 89 | 199.49 | 119 | 199.05 | 149 | 199.01 |
| 30 | 199.32 | 60 | 199.44 | 90 | 199.51 | 120 | 198.93 | 150 | 199.46 |

**Step 2: Moving Range (MR) Calculation**

The moving range ${MR}_{i}$​ was calculated for each pair of consecutive measurements:

${MR}_{i}=\left| X_{i}-X_{i-1} \right|$ ($i=2,3,\ldots,150$)

And the calculated moving range values are summarized in **Table A4**.

**Table A4**. Calculated moving range (${MR}_{i}$) values based on the random sample in Table A3.

| Measure-  Ment No. | Moving Range  (cGy) | Measure-  Ment No. | Moving Range  (cGy) | Measure-  Ment No. | Moving Range  (cGy) | Measure-  Ment No. | Moving Range  (cGy) | Measure-  Ment No. | Moving Range  (cGy) |
| --- | --- | --- | --- | --- | --- | --- | --- | --- | --- |
| 1 | / | 31 | 0.50 | 61 | 0.29 | 91 | 0.04 | 121 | 0.58 |
| 2 | 0.85 | 32 | 0.85 | 62 | 0.03 | 92 | 0.58 | 122 | 0.37 |
| 3 | 0.87 | 33 | 0.17 | 63 | 0.54 | 93 | 0.46 | 123 | 0.62 |
| 4 | 0.31 | 34 | 1.00 | 64 | 0.67 | 94 | 0.10 | 124 | 0.52 |
| 5 | 0.04 | 35 | 0.06 | 65 | 0.96 | 95 | 0.20 | 125 | 0.86 |
| 6 | 0.16 | 36 | 0.56 | 66 | 1.49 | 96 | 0.47 | 126 | 0.48 |
| 7 | 0.25 | 37 | 0.45 | 67 | 0.90 | 97 | 0.30 | 127 | 2.66 |
| 8 | 0.23 | 38 | 3.68 | 68 | 0.34 | 98 | 1.33 | 128 | 3.46 |
| 9 | 0.73 | 39 | 3.13 | 69 | 0.16 | 99 | 1.02 | 129 | 0.60 |
| 10 | 0.20 | 40 | 0.59 | 70 | 0.13 | 100 | 0.33 | 130 | 0.03 |
| 11 | 0.51 | 41 | 1.14 | 71 | 0.77 | 101 | 0.05 | 131 | 0.38 |
| 12 | 0.52 | 42 | 0.16 | 72 | 1.29 | 102 | 0.60 | 132 | 0.35 |
| 13 | 1.02 | 43 | 0.28 | 73 | 0.74 | 103 | 1.48 | 133 | 0.21 |
| 14 | 0.15 | 44 | 0.11 | 74 | 0.23 | 104 | 0.27 | 134 | 0.51 |
| 15 | 0.70 | 45 | 0.53 | 75 | 1.09 | 105 | 1.52 | 135 | 0.35 |
| 16 | 0.39 | 46 | 0.63 | 76 | 1.29 | 106 | 0.31 | 136 | 0.38 |
| 17 | 0.93 | 47 | 0.48 | 77 | 0.66 | 107 | 0.32 | 137 | 0.60 |
| 18 | 0.75 | 48 | 1.92 | 78 | 0.53 | 108 | 0.13 | 138 | 0.94 |
| 19 | 1.15 | 49 | 1.62 | 79 | 0.80 | 109 | 0.03 | 139 | 1.41 |
| 20 | 0.87 | 50 | 0.49 | 80 | 0.17 | 110 | 0.81 | 140 | 0.22 |
| 21 | 0.31 | 51 | 0.38 | 81 | 0.12 | 111 | 0.43 | 141 | 0.29 |
| 22 | 0.49 | 52 | 0.65 | 82 | 0.55 | 112 | 0.78 | 142 | 0.43 |
| 23 | 0.06 | 53 | 0.61 | 83 | 0.18 | 113 | 0.88 | 143 | 0.62 |
| 24 | 0.84 | 54 | 0.17 | 84 | 0.92 | 114 | 1.74 | 144 | 0.26 |
| 25 | 0.85 | 55 | 0.37 | 85 | 0.11 | 115 | 1.14 | 145 | 0.76 |
| 26 | 0.04 | 56 | 0.86 | 86 | 0.18 | 116 | 0.24 | 146 | 0.69 |
| 27 | 0.05 | 57 | 0.36 | 87 | 0.98 | 117 | 0.45 | 147 | 0.96 |
| 28 | 0.26 | 58 | 0.15 | 88 | 0.38 | 118 | 0.61 | 148 | 0.29 |
| 29 | 0.27 | 59 | 0.94 | 89 | 0.10 | 119 | 0.70 | 149 | 0.22 |
| 30 | 0.37 | 60 | 0.28 | 90 | 0.02 | 120 | 0.12 | 150 | 0.45 |

**Step 3: Average Moving Range**

The average moving range ($\bar{MR}$) was calculated as:

$\bar{MR}=\frac{\sum_{i=2}^{N} {MR}_{i}}{N-1}=\frac{1}{149}\sum_{i=2}^{150} {MR}_{i}=0.62$ (cGy)

**Step 4: Central Line (CL)**

The central line (process mean) was calculated as:

${CL}_{X}=\bar{X}=\frac{\sum_{i=1}^{N} X_{i}}{N}=\frac{1}{150}\sum_{i=1}^{150} X_{i}=199.19$ (cGy)

**Step 5: Proportion of Positive Deviations (**$\boldsymbol{P}_{\boldsymbol{X}}$**​)**

The proportion of data points above the mean was calculated as:

$$P_{X}=\frac{\sum_{i=1}^{N} I\left( \bar{X}-X_{i} \right)}{N}=\frac{79}{150}=0.527$$

**Step 6: Determination of** $\boldsymbol{d}_{\boldsymbol{2}}^{\boldsymbol{WSD}}$

The value of $d_{2}^{WSD}$​ corresponding to the calculated $P_{X}$ (0.527) was determined by directly referencing the tabulated values, as $P_{X}\geq0.5$. Linear interpolation between adjacent values was applied if an exact match was not available. Since $P_{X}$ fell between 0.52 (with $d_{2}^{WSD}$=1.126) and 0.54 (with $d_{2}^{WSD}$=1.121), linear interpolation was performed, resulting in a final $d_{2}^{WSD}$​ value of 1.124**.**

**Step 7: Control Limit Calculation**

The LCL and UCL were calculated as:

${LCL}_{X}=\bar{X}-\frac{3\cdot\bar{MR}}{d_{2}^{WSD}}{\cdot2\left( 1-P \right.}_{X})=199.19-\frac{3\times0.62}{1.124}\times2\times(1-0.527)=197.62$ (cGy)

${UCL}_{X}=\bar{X}+\frac{3\cdot\bar{MR}}{d_{2}^{WSD}}{\cdot2P}_{X}=199.19+\frac{3\times0.62}{1.124}\times2\times0.527=200.93$ (cGy)

**Step 8: Identification and Elimination of Out-of-Control Points**

All data points in the random sample were evaluated against the calculated LCL and UCL. Measurements falling outside the control limits were classified as OOC points and excluded from the dataset. For the example in **Table A3**, two measurements—Measurement 38 (196.11 cGy) and Measurement 127 (202.14 cGy)—were identified as OOC points and removed.

**Step 9: Iterative Refinement**

After eliminating OOC points, the control limits (LCL, CL, and UCL) were recalculated based on the remaining data following Steps 2 through 8. This iterative "Identify–Eliminate–Recalculate" procedure was repeated until no further OOC points were detected and the dataset achieved statistical control. The final control limits obtained from this process for this example were: LCL_1_ = 197.81 cGy, CL_1_ = 199.19 cGy, UCL_1_ = 200.73 cGy.

**Step 10: Repetition Across 1,000 Random Samples**

To ensure robustness against sampling variability, the entire procedure described in Steps 1 through 9 was independently repeated 1,000 times.
For each repetition:

(1) A new randomly sampled dataset was generated from the original Phase I data.

(2) The iterative "Identify–Eliminate–Recalculate" process was applied to each sampled dataset.

(3) The final control limits (LCL, CL​, and UCL​) were recalculated after achieving statistical control.

After completing 1,000 iterations, 1,000 sets of control limits—denoted as LCL_j_ ​, CL_j_, and UCL_j_ ​for $j=1,2,\ldots,1000$—were obtained for each QA metric. The **median** values across these sets were then adopted as the final control limits, serving as the tolerance limits for the corresponding metric.

For the daily output dose at gantry 0°, the final tolerance limits determined were: LCL = 197.85 cGy, CL = 199.19 cGy, UCL = 200.71 cGy (**Table A5**). An individual control chart constructed using these final tolerance limits is illustrated in **Figure A1**.

**Table A5**. Statistical summary of control limit parameters and process characteristics derived from 1,000 randomized iterations.

|  | Times | Mean | Standard deviation | Min | Max | **Median** |
| --- | --- | --- | --- | --- | --- | --- |
| $P_{X}$ | 1000 | 0.53 | 0.03 | 0.45 | 0.62 | 0.53 |
| $d_{2}^{WSD}$ | 1000 | 1.12 | 0.01 | 1.07 | 1.13 | 1.12 |
| LCL (cGy) | 1000 | 197.85 | 0.11 | 197.42 | 198.15 | 197.85 |
| CL (cGy) | 1000 | 199.19 | 0.04 | 199.08 | 199.33 | 199.19 |
| UCL (cGy) | 1000 | 200.72 | 0.13 | 200.25 | 201.28 | 200.71 |
| OOC Points | 1000 | 2.02 | 1.39 | 0.00 | 8.00 | 2.00 |


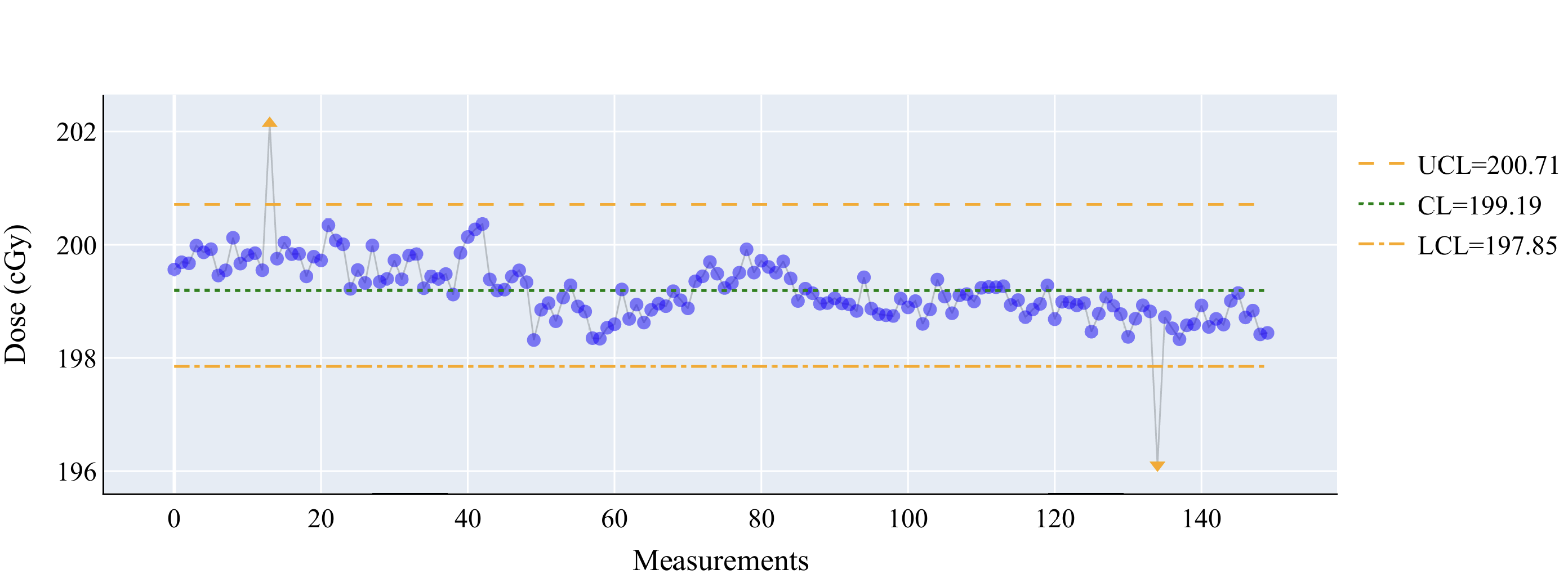


**Figure A1**. Final individual control chart (I-Chart) for the daily output dose at gantry 0°, constructed using the established tolerance limits.

**Reference**

1. Chang YS, Bai DS, Chang YS, Bai DS. Control charts for positively‐skewed populations with weighted standard deviations. Qual Reliab Eng Int. 2001;17:397–406.
